# Diatomaceous Earth for Arthropod Pest Control: Back to the Future

**DOI:** 10.3390/molecules26247487

**Published:** 2021-12-10

**Authors:** Valeria Zeni, Georgia V. Baliota, Giovanni Benelli, Angelo Canale, Christos G. Athanassiou

**Affiliations:** 1 Department of Agriculture, Food and Environment, University of Pisa, via del Borghetto 80, 56124 Pisa, Italy; valeriazeni93@gmail.com (V.Z.); angelo.canale@unipi.it (A.C.); 2 Laboratory of Entomology and Agricultural Zoology, Department of Agriculture, Crop Production and Rural Environment, University of Thessaly, Phytokou Str., 38446 Volos, Greece; mpaliota@agr.uth.gr (G.V.B.); athanassiou@agr.uth.gr (C.G.A.)

**Keywords:** urban pests, agricultural pests, aphids, cockroaches, kissing bugs, insect vectors, green insecticides, mosquitoes, moth pests, non-target toxicity, stored product pests, termites

## Abstract

Nowadays, we are tackling various issues related to the overuse of synthetic insecticides. Growing concerns about biodiversity, animal and human welfare, and food security are pushing agriculture toward a more sustainable approach, and research is moving in this direction, looking for environmentally friendly alternatives to be adopted in Integrated Pest Management (IPM) protocols. In this regard, inert dusts, especially diatomaceous earths (DEs), hold a significant promise to prevent and control a wide range of arthropod pests. DEs are a type of naturally occurring soft siliceous sedimentary rock, consisting of the fossilized exoskeleton of unicellular algae, which are called diatoms. Mainly adopted for the control of stored product pests, DEs have found also their use against some household insects living in a dry environment, such as bed bugs, or insects of agricultural interest. In this article, we reported a comprehensive review of the use of DEs against different arthropod pest taxa, such as Acarina, Blattodea, Coleoptera, Diptera, Hemiptera, Hymenoptera, Ixodida, Lepidoptera, when applied either alone or in combination with other techniques. The mechanisms of action of DEs, their real-world applications, and challenges related to their adoption in IPM programs are critically reported.

## 1. Introduction

Among different types of inert materials currently adopted in pest control, diatomaceous earths (DEs) hold a prominent position, as they are apparently the most often tested material for this purpose. A search in *Journal of Stored Products Research* for published papers between January 2019 and January 2021 revealed the publication of 13 papers with “diatomaceous earth” on their title, emphasizing the utilization of DEs in stored product protection. DEs are not only used for the management of insects and other arthropods, but they also have multiple uses including the control of different pathogens, such as fungi and bacteria [1,2,3,4]. Other types of inert dusts, such as zeolites [5] or kaolin [6], have been also investigated for pest control. This work will be focused solely on the use of DEs in crop protection but also in post-harvest and urban pest control, highlighting their wide applicability.

In a recent review paper, Athanassiou et al. [7] categorized the materials that can be used in pest control and fall into the category of “nano” under the general term of nanoparticles. Although there are cases where DE particles can touch the “nano” scale, DEs are generally classified in the “micro” category and can be considered as “microparticles” in contrast with nanoparticles.

DEs are the fossilized remains of phytoplankton, which are diatoms that occurred mostly during the Miocene and Eocene periods [1]. Diatoms are unicellular eukaryotic algae that are characterized by an external skeleton (frustule) rich in silicon dioxide whose fossilized remains constitute DEs [1,2,8]. These diatoms are abundant either in fresh-water or marine environments, but they are also present in terrestrial ecosystems. 

The present review provides a focus on the utilization of DEs to manage different arthropod pest categories when applied either alone or in combination with other techniques.

## 2. Which Is the Mode of Action of DEs?

There are different theories about the insecticidal effect of DEs [2]. It is generally considered that DE particles attach to the insects’ cuticle, causing death through desiccation [1,2,9], although the abrasion is also a complementary action, i.e., through cuticular micro-wounds [2]. The shape of DEs may be a critical factor in this sense, as round-shaped diatom may lead to more rapid water absorption, while sharp-shaped DE acts more as an abrasive factor [1,2,9,10]. Nevertheless, the shape of the diatom, and probably its action (i.e., sorption vs. abrasion) can be chanced through different processing techniques [11].

## 3. Why Use DEs for Arthropod Pest Control?

Thanks to their characteristics, the use of DEs is advantageous for several types of applications [2,8]. First, DEs are natural substances, and given their low toxicity to mammals and the environment, the registration process is greatly simplified. In addition, being inert materials, DEs have no interaction with the commodity and can be easily removed through standard processing, such as sieving [1,10,12,13,14], while their presence in the final product, such as flour or semolina, does not alter baking or pasta-making properties [1,12]. For more than two decades, DEs have been used as feed additives and in veterinary pest control [1]. Moreover, DEs are easily accessible [8,15]. The natural deposits from where DEs are extracted are found almost everywhere. Following their extraction, these powders are sieved to obtain a homogeneous mixture of particle sizes and dried at approximately 2–6% moisture content [1,11,15]. Finally, due to their mechanism of action, no physiological pest resistance is expected to occur, while tolerance may be exhibited through reduced contact with the DE particles [16,17,18].

## 4. Any Dark Facets for DEs Use in Pest Control?

In general, to be effective, DEs must be applied at elevated concentrations, which are much higher than those of conventional insecticides and often exceed 1000 ppm [2,8,19,20]. In this way, they create a “dusty” appearance on the products and might cause health problems to workers, such as respiratory disorders [1,2,8,10]. In addition, their application on stored products results in the reduction of the test weight (weight to volume ratio), which is a critical characteristic in the international grain market [1,12]. 

In this scenario, we focused on current knowledge and challenges on the use of DEs in stored products as well as for managing arthropod pests of agricultural importance, urban pests, and vectors of public health relevance. The potential impact of DEs on non-target species is also discussed.

## 5. DEs to Control Stored Product Pests

Currently, most studies assessing the toxicity of DEs on arthropods of economic importance are focused on stored product pests. Storing durable commodities is significant since it ensures stable food and feed production all year long and on a global scale. However, the storage environment, which may range from warehouses to retail shelves, is also a prosperous place for a range of insects to thrive [21]. Insect infestations have multiple effects on stored food, feed commodities, and seeds. Beyond the direct damage caused by food consumption, insects also pose a quarantine threat. Insect fragments within durable edible products provoke allergic reactions, alter the organoleptic characteristics, and potentially carry disease-causing pathogens [22]. Therefore, even a small percentage of damage may result in profound monetary losses. Despite the technological advantages over the years, most segments of the food industry are very susceptible to insect infestations, especially when it comes to stored grains [23]. On the other hand, pest management currently depends mostly on chemical methods, but such approaches must be at least improved by adopting more sustainable and eco-friendly treatments for raw and processed commodities [24]. Herein, we analyze the various factors routing the efficacy of DEs against stored product pests and their real-world use, even in combination with fungal and plant-borne pesticides.

### 5.1. Biotic and Abiotic Factors That Influence the Efficacy of DEs

Given their high absorptive power, the efficacy of DEs is highly determined by the levels of relative humidity (R.H.)/moisture content (m.c.). Hence, in humid conditions, some types of DEs may not be as effective as in dry conditions. For instance, Vayias and Athanassiou [25] tested larvae of the confused flour beetle, *Tribolium confusum* Jacquelin du Val (Coleoptera: Tenebrionidae) for their susceptibility to DEs, and they found that the efficacy of commercial DEs was reduced as the R.H. level rose from 55% to 65%. This is particularly important for grain protection, as R.H. levels between 55% and 75% correspond with an equivalent of 10.5% to 14% m.c., which are realistic ranges for long-term storage [26,27]. However, there are studies where the efficacy of DEs was not much affected by the increased R.H., suggesting that certain DE types do not interact much with moisture. A slurry formulation of DE, i.e., a mixture of DEs and water, may not be as effective as dust (powder) formulations [28]. However, a slurry formulation can be more practical in terms of direct application in the commodity with the same technology as traditional grain protectants [28,29].

The temperature might act indirectly on the efficacy of DEs, since at a higher temperature, the water loss occurs faster. In addition, insect mobility is increased at elevated temperatures, causing an increase in the contact with the DE particles. Athanassiou et al. [20] tested a commercially available DE on wheat for the control of adults of *T. confusum* and the rice weevil, *Sitophilus oryzae* (L.) (Coleoptera: Curculionidae), and noticed that there was a positive correlation between the mortality rates and the temperature. Indeed, by increasing the temperature by 10 °C, Athanassiou et al. [20] reported that the mortality rates were raised from approximately 45% at 22 °C to 100% at 32 °C. Other studies show similar results for a wide range of species [2,25,30,31], but some reports show that the increase in temperature decreases mortality [30,32]. The type of commodity on which DEs are applied is another critical aspect that should be considered. In the case of stored grain protection, not all grains are equal in terms of their response to DEs, suggesting that there are specific interactions with the external parts of the grains mass that may partially inactivate the DE particles. In a series of studies [13,14,19] it was shown that DEs are less effective on maize than on small grains, such as wheat, rice, and barley. Kavallieratos et al. [13] used sieves to remove two different DEs from eight grains, and the percentage of DEs removed was always higher on maize and minimal on wheat or barley. In addition, DE adherence was much lower in peeled barley than in non-peeled barley, which is a clear indication that the shape of the external kernel part is critical in maintaining the DE particles [13]. Still, these adherence differences among the different grains did not correlate with adult mortality in the lesser grain borer, *Rhyzopertha dominica* (F.) (Coleoptera: Bostrychidae) [13,14].

Different target species have different levels of susceptibility to DEs. It is generally expected that soft-bodied insects are more vulnerable to DEs, as their cuticula can be easily damaged, causing rapid desiccation [29]. However, this is not always true. For instance, stored product mites, such as Astigmata, are extremely vulnerable to DEs, which is considered as a direct consequence of their sensitivity to water loss and their thin cuticles [29,33]. Nevertheless, another category of soft-bodied stored product pests, psocids (Psocoptera), are extremely tolerant to DEs [34]. Psocids have a certain mechanism that can moderate water loss and absorb moisture from the air to compensate losses [35,36]. Larvae are considered more susceptible to DEs than adults [29]. For instance, Vayias and Athanassiou [25] found that *T. confusum* larvae were more susceptible to DE than adults, with early-stage larvae being the most vulnerable larval instar. However, this is not true for the yellow mealworm, *Tenebrio molitor* L. (Coleoptera: Tenebrionidae), where adults are susceptible to DEs, but larvae remain unaffected due to the occurrence of a mechanism that moderates water loss [37]. The adults of the red flour beetle, *Tribolium castaneum* (Herbst) (Coleoptera: Tenebrionidae) and *T. confusum*, are being considered as the least susceptible beetle species to DEs, with the latter slightly more tolerant [19,25,29,38,39]. On the other hand, adults of the rusty grain beetle, *Cryptolestes ferrugineus* (Stephens) (Coleoptera: Laemophloeidae), are very susceptible to DEs, as they are flat-bodied, and water loss can rapidly occur [1,9,30]. Still, there are dissimilar and not directly comparable results for different species of stored products [15,29,30], but some general conclusions can be drawn based on the above observations. Apart from body size, shape, and characteristics, insect mobility is a critical parameter, as slow-moving insects may have a lower DE particle uptake. This is considered a key feature for the reduced susceptibility of *R. dominica* to DEs [1,30], although some reports show that this species is particularly susceptible to different DEs [13,14].

Some additional parameters that influence the efficacy of DEs have to do with their physicochemical characteristics. For instance, it has been shown that particle size is an important parameter, and the smaller the particles, the highest the DE efficacy against insects [1,8,9,11]. Vayias et al. [9] have shown that DEs with particles that were smaller than 45 μm were more effective than DEs with larger particles against *R. dominica*, *S. oryzae,* and *C. ferrugineus*. Nonetheless, Baliota and Athanassiou [11] have shown that it is the particle shape, rather than the size, that had a certain effect on the insecticidal value of DEs, and that smaller particles do not necessarily mean higher efficacy. Moreover, very small particles may not be desirable for safety issues [2].

Several physicochemical characteristics can be further utilized toward the prediction of the expected insecticidal value of DEs. Korunić [10] summarized these characteristics in standardized testing, which can be carried out for rapid screening of DE samples, without the need to conduct bioassays with insects, which is a time-consuming procedure. The silicon dioxide content and pH are important factors, while clay and other impurities are not desirable [10]. Even more important parameters are the tapped density, the bulk density reduction, and the adherence to grain kernels [1,10]. Diatom species, origin, and other characteristics may be less important [1,2,9,11,15,40].

### 5.2. Combinations with Contact Synthetic Insecticides

One of the possible solutions to the implications caused by the high doses of DEs is the combination of DEs with other substances thanks to the adsorptive nature of the DE particles. Indeed, the utilization of DEs as a carrier is a promising solution not only for the application of insecticides in reduced concentrations but also to combine at least two different modes of action, i.e., desiccation through the inert dusts and an additional action depending on the type of chemical (e.g., neurotoxic, etc.). Several studies have indicated a significant potential and even synergism of combinations of commercial DE formulations with residual insecticides. Wakil et al. [41] reported high mortality rates of *R. dominica* in wheat, rice, and maize treated with a combination of thiamethoxam and a commercial DE formulation, SilicoSec^®^ (Biofa GmbH, Munsingen, Germany), in relatively low doses (0.25, 0.5, and 0.75 ppm for thiamethoxam and 100 ppm for SilicoSec^®^). The combination of 150 ppm of Protect-It^®^ (Hedley Technologies Inc., Mississauga, ON, Canada) with 1.25, 2.5, or 5.0 ppm of imidacloprid resulted in higher mortality rates of different stored product insects than applications of these insecticides alone at almost all exposure intervals and commodities tested [42]. Ceruti and Lazzari [43] used 500 and 1000 ppm of Keepdry^®^ (Irrigação Dias Cruz ME, Brazil) in combination with 0.5 or 1.0 g a.i./t of deltamethrin powder, which may represent an efficient control measure against the maize weevil *Sitophilus zeamais* Motschulsky (Coleoptera: Curculionidae) in stored corn, highlighting the potentials of having reduced residues of deltamethrin, as compared with using this active ingredient alone. Arthur [44] stated that an insecticide formulation (F2) containing 0.03% deltamethrin, 0.37% piperonyl butoxide, 0.95% chlorpyriphos-methyl, 10% mineral oil, and 88% Protect-It^®^ was extremely effective in wheat, maize, and paddy rice at the rate of 100 ppm against *S. oryzae*, *S. zeamais*, *R. dominica* and *T. castaneum*. Awais et al. [45,46] tested three different doses of the DE formulation Concern (Wood Stream^TM^ Corporation, Lititz, PA, USA) combined with the Insect Growth Regulators (IGRs) lufenuron and tebufenozide against *T. castaneum* and the khapra beetle, *Trogoderma granarium* Everts (Coleoptera: Dermestidae) respectively, with the overall conclusions to specify that the combined use of DEs and IGRs is highly operative and beneficial for stored product insect control. A combination of the IGR S-methoprene and Protect-It^®^ could also be a promising mixture as reported by Arthur [47]. In that study, the mixture had an additive effect and reduced the concentrations of both components required to suppress the progeny of *R. dominica* compared to the application of each insecticide alone [47]. In addition, SilicoSec^®^ (25 ppm) and beta-cyfluthrin (0.125 or 0.25 ppm) acted synergistically for the control of *T. castaneum* and, especially, *S. oryzae* [48]. The long-term protection of a given insecticide is one of the key elements in stored-grain pest management, aiming to prevent new infestations and control the reproduction of the already existing individuals. Mixtures with DEs have the potential to enhance the residual efficacy of an insecticide. Wakil et al. [49] reported an increased mortality of adults of *R. dominica* over 9 months of wheat storage with applications of 200 ppm of SilicoSec^®^ and 0.5 ppm thiamethoxam in comparison with the residual efficacy of thiamethoxam alone, which was decreased significantly 2 months after its application. Korunić et al. [50] applied a formulation containing a low quantity of DE and small amounts of deltamethrin and reported a high residual efficacy against *S. oryzae*, *R. dominica*, and *T. castaneum* even 12 months after the treatment. Wakil and Schmitt [51] also found that applications with 150 ppm of DEBBM (DE + bitterbarkomycin) plus 5.0 ppm imidacloprid were more effective than single insecticidal treatments for a period of five months, against all tested species on stored wheat.

### 5.3. Combination with Fungal Agents 

Recently, extensive research focused on the adoption of entomopathogenic fungus species as an alternative approach to control insect pests of stored grain [52,53,54,55,56,57]. Fungal species such as *Beauveria bassiana* (Balsamo) Vuillemin (Ascomycota: Hypocreales), which is probably the most examined entomopathogenic fungus for stored product insects [58,59,60,61,62,63,64] has a complex interaction with cuticular lipids [65]. Results exalted the suitability of fungi as stored-product protectants but also pointed out their need for peculiar humid conditions to achieve satisfactory conidial adherence, germination, and penetration through the cuticle [66,67,68]. Increased humidity in stored commodities should be avoided [29], and hence, the fungal strains should be effective at drier conditions. Since DEs best perform under low humidity levels [2,25], the combination of fungi with DEs is very promising. The synergistic effect between DEs and entomopathogenic fungi expands the area for fungal spore penetration, increasing insect mycosis [40,62,69,70,71,72,73]. In addition, Batta [71] reported that the utilization of two different formulations of DE dusts, i.e., The Fossil Shield 90.0^®^ (The Fossil Shield Co., Eiterfeld, Germany) and SilicoSec^®^ (Agrinova GmbH, Obrigheim/Muhlheim, Germany), had a negligible effect on the viability of conidia of two fungal species. Dal Bello et al. [74] indicated the DE–fungal combinations to overcome some of the constraints in the use of fungi as biocontrol agents.

Applications of mixtures with these two ecologically compatible agents is a very appealing approach to IPM and can grant a more consistent management of multiple pest species under a wider range of environmental conditions.

The study of Athanassiou and Steenberg [70] demonstrated the potentials of these two agents together. The authors tested the insecticidal effect of *B. bassiana* combined with relatively low doses of Insecto^®^ (Insecto Natural Products Inc., Costa Mesa, CA, USA), SilicoSec^®^, and PyriSec^®^ (Biofa Gmbh, Germany), reporting a high level of control against the granary weevil *Sitophilus granarius* (L.) (Coleoptera: Curculionidae) under a broad range of temperatures and relative humidity levels [70]. In another published work by Wakil et al. [73], the application of 15 and 30 ppm of DEBBM combined with three doses of *B. bassiana* considerably increased adult mortality of *R. dominica*, especially at increasing temperatures and longer exposure intervals compared with DEBBM and *B. bassiana* alone. The synergistic interaction between Protect-It^®^ and *B. bassiana* against several major stored-product insect species was also proved in laboratory bioassays [65,75]. Shafighi et al. [76] mentioned the high “speed of kill” of the combination of low doses of SilicoSec^®^ when combined with entomopathogenic fungi against *T. castaneum.* Rizwan et al. [77] reported that the combination of the commercial DE formulation Diafil 610 (Celite Corporation, Lompoc, CA, USA) with *B. bassiana* had a suppressive effect on progeny (F1) production of the same beetle species. In field trials conducted on small farms, the treatment with mixtures of DE and *B. bassiana* outperformed the analogous combinations with imidacloprid after six months of storage [51]. 

*Metarhizium anisopliae* (Metschnikoff) Sorokin (Deuteromycotina: Hyphomycetes) and *Paecilomyces fumosoroseus* (=*Isaria fumosorosea*) (Wise) Brown & Smith (Ascomycota: Hypocreales) have also become a test subject for their insecticidal efficacy when combined with DEs, with reports to be in accordance with their potentials as control agents against several insect species, providing also long-term protection when applied in a variety of stored grains [40,61,76,78,79]. The virulence of *P. fumosorosea* integrated with DEBBM was shown to be an effective control measure for *R. dominica* in stored wheat [80]. *Nomuraea rileyi* (Farl.) Samson (Ascomycota: Hypocreales) and *Lecanicillium lecanii* (Zimm.) Zare & W.Gams (Ascomycota: Hypocreales) along with natural or modified DE formulations have been reported to show insecticidal, repellent, and ovicidal effects against *Bruchidius incarnatus* (Boheman) (Coleoptera: Chrysomelidae) and *R. dominica* under a variety of temperature and relative humidity conditions [81]. 

### 5.4. Combination with Botanicals

Plant extracts, essential oils, and other plant-based products are all ingredients with the potential to control stored-product insects [82,83]. However, their utilization is sometimes challenging due to their instability and high recommended doses. Combining them with DEs may enhance their properties, pursuing better insecticidal performances at lower doses and under a wide range of conditions. Several studies have been conducted toward this direction, using compounds from several sources. Bitterbarkomycin (BBM), a plant extract from the roots of *Celastrus angulatus* Max (Celastraceae), is known for its strong insecticidal and antifeedant activity against several insect species; low doses of DEBBM led to high mortality rates of *S. oryzae, S. zeamais*, *T. castaneum, R. dominica,* and *C. ferrugineus* in stored wheat [28,34]. Two DE formulations enhanced with abamectin, a macrocyclic lactone produced either directly by the actinomycete *Streptomyces avermitilis* or generated through semisynthetic modifications [84], were found to have high insecticidal properties against stored-product insects at rates as low as 75–125 ppm [28].

Constraints of the use of essential oils, such as their poor penetration, strong odor, lack of persistence, and high concentration requirements could be reduced if combined with DEs. Yang et al. [85] tested a combination of essential oil derived from *Allium sativum* L. (Amaryllidaceae) with 250 ppm of a DE formulation, reporting a strong synergistic effect and high initial efficacy against *S. oryzae*. Ziaee et al. [86] examined the synergistic/antagonistic interaction between *Carum copticum* (L.) (Apiaceae) essential oil with natural DE formulations of Iranian deposits against *T. confusum* and *S. granarius*, reporting the potentials of the combination for use in IPM programs. The same authors also stated that the essential oil increased the DE efficacy by increasing insect’s locomotion activity through the particles and, at the same time, DEs reduced the oil concentration for the satisfactory protection of stored products. A new insecticide formulation using Celatom MN 23 (Celatom Diatomaceous Earth Functional Additives Technical Data Sheet, EP Minerals, Reno, NV, USA) enhanced with essential oil extracted from *Anethum graveolens* L. (Apiaceae) has been also examined by Korunić and Fields [87] and found to be effective in controlling four stored-product beetle species at lower doses and with far fewer negative effects on bulk density than using the DE alone. On the contrary, Campolo et al. [88] reported an antagonistic effect of *Citrus sinensis* (L.) Osbeck (Rutaceae) peel essential oil when admixed with the DE formulation Protector (Intrachem Bio, Grassobbio, Lombardy, Italy). Paponja et al. [89] developed an enhanced DE formulation admixing SilicoSec^®^ with several botanicals (essential oil lavender, corn oil, and bay leaves dust) and silica gel, reporting higher mortality of all three insect species tested. Successful formulations of DEs and botanicals for the control of storage pests may be expected soon, but further testing is required to determine the duration of efficacy, cost of formulations, testing for their effect on non-target organisms, human safety, and effects on end-use quality.

## 6. DEs and Their Application in Urban, Agricultural, and Medical Environments

In the following paragraphs, we reviewed the studies that have investigated the efficacy of DEs against urban, medical, and agricultural pests [90,91,92]. Against these important pest groups, the insecticidal activity of DEs has been examined both when applied alone or in combination with entomopathogenic fungi or botanicals [42,93], following the same approach shown in the above-reported paragraphs dealing with stored product pest control. As a general trend, it has been noted that the biological activity of DEs increased when combined with entomopathogenic fungi [42,94].

### 6.1. DEs to Control Urban Pests

Insects and mites have successfully adapted over the years to the urban environment thanks to their ability to utilize food resources and harborages with humans [91]. These arthropod species can also transmit pathogenic organisms to food, as well as damages to house structures [91,95,96,97]. The control of arthropod vectors and pests, including urban ones, is challenging because of their strong reproductive ability, adaptability, and growing resistance to insecticides [98]. In addition, the adoption of insecticides in indoor areas is hazardous for human health [91]. Recently, several studies investigated the adoption of DE as an alternative to insecticides, highlighting their efficacy on different urban pests through different application scenarios [91,96,99,100,101,102,103] (Table 1). 

The efficacy of DEs has been widely investigated on cockroaches, which are a worldwide public health pest that causes water and food contamination through transmitting pathogens mechanically, such as different forms of gastroenteritis [91,96]. A study compared the mortality of adult males and nymphs of the German cockroach, *Blattella germanica* L. (Blattodea: Ectiobidae), when DEs are applied as dry formulations or with the addition of water [96]. Mixing DE with water reduced the DE effectiveness, and the LC_50_ value was found to be 10 times lower if compared with dry DEs [96]. Similar results have been also found in stored product pests treated with dry DEs or with DEs formulated in water [30]. Overall, the bioactivity of DEs is inversely proportional to the water content and relative humidity [1]. To overcome the limitations related to high relative humidity conditions, mixing DEs with highly hydrophobic silanes may be a solution [99]. As reported by Faulde et al. [99], when DEs are mixed with hydrophobic silanes, a complete control of *B. germanica* could be achieved under humid conditions (R.H. > 80%) within 11 days [99]. In this work, it has been reported that the highest mortality rate of *B. germanica* (100% after 110 h) was achieved with the commercial DE Fossil-Shield^®^ 90.0 S White, whose hydrophobicity increased by 3% Aerosil^®^ with 1,1,1-trimethyl-*N*-trimethyl silane [99]. The same modified DEs led to the complete eradication of American cockroach, *Periplaneta americana* L. (Blattodea: Blattidae), and the silverfish, *Lepisma saccharina* L. (Thysanura: Lepismatidae), within 10 days, but the complete population suppression was not achieved in the case of the oriental cockroach, *Blatta orientalis* L. (Blattodea: Blattidae) [100]. These results highlight that cockroach susceptibility to DEs not only varies according to its formulations and their modifications, but it is also species-dependent [99,100]. Overall, hydrophobized DEs are more effective on certain cockroach species because of the higher absorption capacity of their cuticular waxes and the subsequent death by desiccation [99]. 

Thanks to their properties, DEs may act as physical barriers for arthropod pest intrusions and can be used to forecast the occurrence of subterranean termites that threaten housing construction and forest trees [101]. A study conducted by Gao et al. [101] showed that *Reticulitermes chinensis* Snyder (Rhinotermitidae: Blattodea) adult workers were not able to penetrate a 3 mm layer of dry DEs, suppressing their tunneling behavior, and died as a consequence of their movement. As reported by Ahmed et al. [103], mixing the soil with biofertilizers and DE increased the mortality and reduced the gallery length of another subterranean termite species, *Coptotermes heimi* (Wasmann) (Rhinotermitidae: Blattodea). On the other hand, DEs cannot be used as a barrier to prevent penetration of the soil surface by *Coptotermes formosanus* Shiraki (Rhinotermitidae: Blattodea), who was fully able to penetrate a DEs layer in laboratory bioassays [104]. Interestingly, although highly effective for the control of subterranean termites and cockroaches, DEs do not seem to be the most efficient inert dusts to control the pharaoh ant, *Monomorium pharaonis* (L.) (Hymenoptera: Formicidae)—a notorious domestic pest, for which the adoption of chemical-based insecticides is not recommended, particularly when ants infest crowded buildings such as hospitals [105]. Van Den Noortgate et al. [106] highlighted that the efficacy of DEs was lower if compared with various porous powders. For instance, zeolites ordered mesoporous silica material, and carbon black performed better than the DE benchmark material, especially the activated carbon powder (ACP) whose survival median time was almost four times shorter than that of the DEs (LT_DE_: 95 min; LT_ACP_: 25 min) [106].

**Table 1:** Local and commercial diatomaceous earths (DEs) evaluated against immature and adult stages of arthropods of urban interest. In addition to the mortality rates, the SiO_2_ content (%) and the diameter of particles (μm) are reported. NA = not available data.

Pest Species	Family	Order	Developmental Stage	Tested DE	SiO_2_ Content (%)	Ø Particles (µm)	Formulation	Mortality Rates	References	Notes
*Blatta* *lateralis*	Blattidae	Blattodea	Nymph	Turco 000	83–95	1–10	Dry	>90% after 12 h	[91]	Local commercialized DEs; 1 g/m^2^ of DEs
*Blatta* *lateralis*	Blattidae	Blattodea	Nymph	Turco 004	83–95	10–30	Dry	>90% after 20 h	[91]	Local commercialized DEs; 1 g/m^2^ of DEs
*Blatta* *lateralis*	Blattidae	Blattodea	Nymph	Turco 020	83–95	43–65	Dry	>90% after 24 h	[91]	Local commercialized DEs; 1 g/m^2^ of DEs
*Blatta* *orientalis*	Blattidae	Blattodea	Adult + Nymph	Fossil Shield 90.0 S White^®^	0.35% (*w*/*v*)	5	Dry	70.6% on day 10	[99]	
*Blattella* *germanica*	Ectobiidae	Blattodea	Nymph	NA	NA	NA	Dry	LC_50:_ 4.2380 g/m^2^^(^*^)^ LC_50_: 5.2148 g/m^2^^(^**^)^ LC_50_: 12.9034 g/m^2 ^^(^***^)^	[96]	
*Blattella* *germanica*	Ectobiidae	Blattodea	Adult (♂)	NA	NA	NA	Dry	LC_50:_ 8.0307 g/m^2^ LC_90_: 167.7116 g/m^2^	[96]	No report if the LC_50/90_ were at 24 h, 48 h, or 72 h
*Blattella* *germanica*	Ectobiidae	Blattodea	Nymph 2nd stage	NA	NA	NA	Dry + Water (50 mL)	LC_50:_ 20.0358 g/m^2^^(^*^)^; LC_50_: 7.9173 g/m^2 ^^(^**^)^ LC_50_: 6.3729 g/m^2 ^^(^***^)^	[96]	
*Blattella* *germanica*	Ectobiidae	Blattodea	Adult (♂)	NA	NA	NA	Dry + Water (50 mL)	LC_50:_ 7.4093 g/m^2^ LC_90_: 91.2063 g/m^2^	[96]	No report if the LC_50/90_ were at 24 h, 48 h, or 72 h
*Blattella* *germanica*	Ectobiidae	Blattodea	Adult	BGN-1 (Local Turkish DEs)	NA	NA	Dry	100% mortality after 2 days (dose 5 g/m^2^ and 10 g/m^2^) on all type of floors	[102]	Ceramic tiles, Concrete floor, and parquet
*Blattella* *germanica*	Ectobiidae	Blattodea	Adult + Nymph	Fossil Shield 90.0 S W	0.35% (*w*/*v*)	5	Dry	100% mortality on day 6	[99]	
*Blattella* *germanica*	Ectobiidae	Blattodea	Adult + Nymph	Diamol KMT SilicoSec^®^ Fossil Shield 90.0^®^ Fossil Shield 90.0 W^®^ Fossil Shield 90.0 S^®^ Fossil Shield 90.0 S W^®^ Fossil Shield 95.0^®^	0.35–0.40 (*w*/*v*)	5–7	Dry	Daily motility: control > Diamol KMT SilicoSec > FS 90.0 > FS 90.0 W > FS 90.0 S = FS 95.0 FS 90.0SW	[100]	
*Coptotermes* *formosanus*	Rhinotermitidae	Blattodea	Adult	Local DE	NA	NA	Dry	38.75% ± 6.60	[104]	No decrease in tunneling behavior
*Coptotermes* *heimi*	Rhinotermitidae	Blattodea	Adult	NA	NA	NA	Dry	At the highest dose of biofertilizer the mortality was lower than 40%	[103]	DEs were added to the soil + biofertilizers
*Lepisma* *saccharina*	Lepismatidae	Thysanura	Adult	Fossil Shield 90.0 S White^®^	0.35% (*w*/*v*)	5	Dry	100% mortality on day 9	[99]	
*Lepisma* *saccharina*	Lepismatidae	Thysanura	Adult	Fossil Shield 90.0 S White^®^	0.35% (*w*/*v*)	5	Dry	Low motility in both control and treated species	[99]	
*Monomorium* *pharaonis*	Formicidae	Hymenoptera	Adult	Lumino^®^	NA	NA	Dry	Lethal time: 95 minutes	[106]	No evidence if LT_50_ or LT_90_
*Periplaneta* *americana*	Blattidae	Blattodea	Adult + Nymph	Fossil Shield 90.0 S White^®^	0.35% (*w*/*v*)	5	Dry	100% mortality on day 8	[99]	
*Periplaneta* *americana*	Blattidae	Blattodea	Adult	K14 (local turkish DEs)	NA	NA	Dry	100% mortality after 11 days (dose 40 g/m^2^) on all type of floors	[107]	Ceramic tiles, Concrete floor, and laminate
*Reticulitermes* *chinensis*	Rhinotermitidae	Blattodea	Adult workers	NA	99	25–45	Moisture and dry DEs	100% after 6 hours when used dried DEs	[101]	10% and 25% of moisture led to low mortality rates Tunneling behavior is reduced in DEs moisture at 10%, 25%, and 50% Worker termites cannot penetrate a 3 mm layer of DEs

(*) LC_50_ calculated at 24 h; (**) LC_50_ calculated at 48 h; (***) LC_50_ calculated at 72 h.

### 6.2. DEs to Control Arthropod Pests and Vectors of Medical and Veterinary Importance

In recent years, several studies have investigated the use of DEs for managing pest and vector species of medical and veterinary importance [90,108,109,110,111,112] (Table 2). Many arthropods can play a pivotal role in the transmission of pathogens and eventually cause diseases in a wide range of vertebrates, including humans, livestock, pets, and wildlife [97]. Herein, we focus on some studies carried out to prove the efficacy of DEs alone or in combination with entomopathogenic fungi against arthropod pests and vectors of public health importance [90,108,109,110,111,112]. Many studies have investigated the adoption of DE to control bed bugs, *Cimex lectularius* L. (Hemiptera: Cimicidae), which are obligatory hematophagous insects that feed commonly on humans [95,97]. Apart from blood sucking, bed bugs are responsible for a range of emotional problems, anxiety, and sleeplessness [90]. As for other urban pests, there is no longer an absolute method to control/eradicate bed bugs, and the management relies either on the use of chemicals, such as pyrethroids, or on non-chemical tools, such as steam [95]. Given their low impact on mammals, DEs recently seem to be more of an option in bed bug control [90,113]. Several commercial DEs are known to be effective in the control of bed bugs, such as Bed Bug Kill [113], DE 51 [108], Mother Earth^®^ D [90,108,114], Alpine^®^ [90,114], Pro-Active^®^, DX13^TM^-dust, and aerosol [90]. As reported by Akhtar and Isman [90], who evaluated the efficacy of several commercial DEs, their activity depends on the content of amorphous silicon dioxide and the dimension of the particles, resulting in the higher effectiveness of one DE to another. The efficacy of DEs may be increased by the addition of a dispersal agent, such as a bed bug alarm pheromone, which enhances bed bug crawling activity, increases bed bug locomotor activity, and thereby causes a higher contact with DE [115]. In addition, DE can be horizontally transferred from a treated bed bug to an untreated one [108]. This phenomenon is typical of gregarious insects and can facilitate the spread of DEs toward spaces that are hard to reach, contributing to the management of public health pests [108]. 

DEs have a recent use in the control of kissing bugs, *Triatoma infestans* (Klug) (Hemiptera: Reduviidae), which is a vector of *Trypanosoma cruzi*, causing Chagas’ disease [97]. To date, several studies emphasize the efficacy of entomopathogenic fungi to control this species [116,117], but low humidity seems to be a limiting factor for fungal infection [117]. In addition, entomopathogenic fungi do not induce quick and high mortality as synthetic insecticides [117]. For these reasons, combining entomopathogenic fungi with oils and DEs may be a solution. The combination of DE + oil eventually enhances the adhesion and spread of particles (DE and conidia) on the lipophilic cuticle. In addition, the fungal development may be favored by the higher moisture provided by the abrasive action of DE and the subsequent trapping of moisture, and lastly, the oil serves as a nutrient source for the fungi [117]. The efficacy of the mixture is already well established, especially against stored-product pests [116,117,118,119,120], as detailed above. In laboratory bioassay, the mixture of *B. bassiana* and a commercial DE caused high mortality rates in all nymph instars and adults of *T. infestans*, ranging from 82% to 100% [120], but the same mixture elicited only 52.4% of *T. infestans* death in a field test in Northern Argentina [116]. Another study highlighted that the efficacy of the commercial DE KeepDry^®^ toward *T. infestans* nymphs is highly increased when combined with vegetable oil and *M. anisopliae* (IP 46)*,* even at a R.H. level of 75% [117]. The same combination was also effective in the control of the yellow fever mosquito, *Aedes aegypti* (L.) (Diptera: Culicidae), a vector or dengue, chikungunya, and Zika virus in the tropical and subtropical regions [109]. Overall, the combination of DE, entomopathogenic fungi, and a mineral/vegetable oil may represent a promising tool for the development of effective management strategies against *T. infestans* and *A. aegypti*. The combination of *M. anisopliae* (IP 119) with the commercial DE KeepDry^®^ has been also successfully evaluated toward the cattle tick, *Rhipicephalus microplus* (Canestrini) (Ixodida: Ixodidae) [121]. The microsclerotia of *M. anisopliae* were incorporated in pellets containing inorganic materials, such as vermiculite, DE, and SiO_2_ [121]. Overall, the pellets formulated with *M. anisopliae* microsclerotia effectively suppressed *R. microplus* in laboratory tests, demonstrating a promising pellet formulation for targeting the non-parasitic stage of this tick on the pasture [121]. Pellets can represent a possible upgrade of conventional granules thanks to their properties: a higher dose uniformity, higher mechanical resistance, narrower particle size distribution, and higher capacity of active incorporation [121]. The combination of fungal spores of *B. bassiana* and commercial DE resulted in significantly increased efficacy against blood-sucking poultry red mite *Dermanyssus gallinae* (De Geer) (Mesostigmata: Dermanyssidae) [94], which is a worldwide hematophagous ectoparasite in poultry farming [122] that is also responsible for the transmission of avian influenza viruses and *Salmonella enterica* ssp. *enterica* (S.) ser. Enteritidis and other important enterobacteria [123,124]. Although DEs were highly effective in the control of *D. gallinae*, Kilping and Steenberg [93] highlighted that four commercial DEs (SilicoSec^®^, Diamol^®^, Protect-It^®^, and Fossil Shield 90.0^®^) elicited avoidance behavior and repellence of the mites on the treated substrate. The authors noticed that the more effective the DE is, the greater the repellent activity. Consequently, the repellent activity had an impact on the efficacy of the inert dusts since mites will avoid treated surfaces. Furthermore, the dry conidia of *B. bassiana* also elicited a repellent response to poultry red mites both when applied on its own and when admixed with a low dose of the commercial DE Diamol [93]. 

In field bioassays, a liquid formulation of DEs elicited high mortality rates of the poultry red mite population [108]. A gradual reduction of the mite population (34% on day 7 to 53.5% on day 14; over 90% on days 21–28) was observed when the application of DE was combined with the mechanical cleaning [108]. The cleaning physically removes the mites and might also help the liquid formulation to stick better to surfaces than when covered with dust [108]. Overall, the adoption of liquid DEs is advisable mainly because product wastes are reduced, and an easier and safer application is provided [125]. Interestingly, DEs were found not to be as efficient as other products, such as kaolin and sulfur, to control the northern fowl mite, *Ornithonyssus sylvarium* (Canestrini & Fanzago) (Mesostigmata: Macronyssidae), which is another threat for hens [125,126]. Testing out the liquid formulations of DE, Martin and Mullens [126] noticed that a significant reduction of the northern fowl mite population occurred when DE was applied for two consecutive weeks, and the highest reduction of mite population was achieved with high concentrations of sulfur (≥5.3%). Although DE effectiveness was found to be lower, in general, their use in dust boxes seems to enhance bird natural dustbathing behaviors, which translates into an increase in animal welfare and a reduction in the use of pesticides [127].

**Table 2:** Local and commercial diatomaceous earths (DEs) evaluated against immature and adult stages of arthropods of medical and veterinary relevance. In addition to the mortality rates, the SiO_2_ content (%) and the diameter of particles (μm) are reported. NA = not available data; IP 46 = entomopathogenic fungus *Metarhizium anisopliae*.

Pest/Vector Species	Family	Order	Developmental Stage	Tested DE	SiO_2_ Content (%)	Ø particles (µm)	Formulation	Mortality Rates	References	Notes
*Aedes* *aegypti*	Culicidae	Diptera	Adults	Keep Dry^®^	86	NA	DE DE + NDE + GIP46 + DE IP46 + DE + NIP46 + DE+ G	DE LT_50_: 10.4 days DE + N LT_50_: 8.9 days DE + G LT_50_: 9.8 days IP46 + DE LT_50_: 5.8 days IP46 + DE + N LT_50_: 5.8 days IP46 + DE+ G LT_50_: 5.9 days	[109]	G: Graxol^®^ (vegetable oil) N: Naturol^®^ (mineral oil)
*Cimex* *lectularius*	Cimicidae	Hemiptera	All stages	Alpine^®^Mother Earth^®^Pro-Active^®^DX13^TM^ dust	NA	NA	Dry	LD_50_ (g m^−2^) Alpine^®^: 4.48 after 24 h LD_50_ (g m^−2^) Mother Earth^®^: 0.18 after 24 h LD_50_ (g m^−2^) Pro-Active^®^: 2.26 after 24 h LD_50_ (g m^−2^) DX13^TM^ dust: 0.17 after 24 h	[90]	DX13^TM^ was horizontally transferred from dead bed bugs to the untreated one
*Cimex* *lectularius*	Cimicidae	Hemiptera	All stages	DX13^TM^ aerosol	NA	NA	Aerosol	Residual mortality (%) DX13^TM^ aerosol: 81% after 21 days (72 h)	[90]	Mortality of bugs on the treated mattress after 32 weeks was 75, 90, and 100% after 24, 48, and 72 h
*Cimex* *lectularius*	Cimicidae	Hemiptera	Adult	DE 51	NA	NA	Dry	LC_50_ (mg) 24.4 and 5.1 at 48 h and 216 h	[108]	LC_50_ was calculated based on the transmission from a treated bug to an untreated one
*Cimex* *lectularius*	Cimicidae	Hemiptera	Nymph + Adult	DE 51	NA	NA	Dry	LC_50_ (mg) unexposed nymph 8.1 LC_50_ (mg) treated adults 6.4	[108]	Treated adults get in contact with untreated nymphs
*Cimex* *lectularius*	Cimicidae	Hemiptera	Nymph/Females	NA	NA	NA	Dry	Low mortality rates	[115]	The addition of alarm pheromone increased the movement of bed bugs throughout the Petri dish
*Cimex* *lectularius*	Cimicidae	Hemiptera	Adult	Bed Bug Killer^®^	NA	NA	Dry	(A) LT_50_ 7.42 days (B) LT_50_ 8.12 days	[113]	(A) Resistant strain (B) Susceptible strain
*Cimex* *lectularius*	Cimicidae	Hemiptera	All stages	Mother-Earth D^®^	NA	NA	Dry	94% of mortality after 10 days	[114]	Alpine^®^ (0.25% Dinotefuran + 95% Diatomaceous Earth) has been also investigated, but its efficacy was lower than DE
*Triatoma* *infestans*	Reduviidae	Hemiptera	Nymphs	Keep Dry^®^	86	NA	DE DE + oil DE + IP46 DE + IP46 + oil	DE: 7.5% after 10 days DE + oil: 5.0% after 10 days DE + IP46: 100% after 10 days DE + IP46 + oil: 100% after 10 days	[117]	Cumulative mortality DE + IP46: LT_50_ = 5.7 days DE + IP46 + oil: LT_50_ = 4.5 days
*Triatoma* *infestans*	Reduviidae	Hemiptera	Eggs	Keep Dry^®^	86	NA	DE DE + oil DE + IP46 DE + IP46 + oil	DE + oil eclosion: 92.5% H.R. 75% DE + oil eclosion: 83% H.R. >98% DE + IP46 + oil eclosion: 95% H.R. 75% DE + IP46 + oil no eclosion H.R. > 98%	[119]	Oil: Graxol^®^
*Triatoma* *infestans*	Reduviidae	Hemiptera	Nymphs	Keep Dry^®^	86	NA	*M. anisopliae* (IP 46) + DE + oil	(a) cumulative mortality: 100% (b) cumulative mortality: 5%	[119]	(a) H.R. > 98%, after 10 days and 24 h of exposition (b) H.R. = 75%, after 10 days and 24 h of exposition
*Triatoma* *infestans*	Reduviidae	Hemiptera	All stages	NA	NA	NA	DE + *B. bassiana*	Nymph 89.5–100%, MLT 5.1–8.3 days Adult 87.5%, MLT 10 days	[120]	MLT = mean lethal time
*Ambloyomma* *americanum*	Ixodidae	Ixodida	Larvae + Nymph	DeadZone	85	NA	Dry	Larval mortality: 100% after 6 h Nymphal mortality: 100% after 24 h	[128]	The DE was compared to a silica-gel based product
*Ambloyomma* *americanum*	Ixodidae	Ixodida	Larvae + Nymph	DeadZone	85	NA	Dry	Larval mortality: 84% after 24 h Nymphal mortality: 44.0% after 24 h	[128]	Highest dose: 10% of DE
*Ceratophyllus* *idius*	Ceratophyllidae	Siphonaptera	All stages	Drione Crawling Insect Killer	NA	NA	Dry	Lower number of fleas in nest treated with DE	[129]	38.12% diatomaceous earth as well as 0.2% pyrethrins and 1.0% piperonyl butoxide
*Dermanyssus* *gallinae*	Dermanyssidae	Acarina	All stages	Fisiocontrol^TM^	86.2	<500	DE in water suspension	Topical mortality 95.4% Residual mortality 97.39%	[111]	Highest dose (10% of DE)
*Dermanyssus* *gallinae*	Dermanyssidae	Acarina	All stages	Fisiocontrol^TM^	86.2	<500	DE + mechanical cleaning	Gradual reduction of mite population, over 90% at days 21–28	[111]	DE dose 10%
*Dermanyssus* *gallinae*	Dermanyssidae	Acarina	All stages	PosturaSec^®^	86.2	200	DE in water suspension	Immature stages: 98.9% (both doses) Adults: 98.8% (5% DE) 100% (10% DE)	[110]	
*Dermanyssus* *gallinae*	Dermanyssidae	Acarina	All stages	Silicosec^®^Ewazid^®^ Silgur F46FS^®^ Istant WFS 90.0 W	NA	NA	DE in water suspension	Silicosec^®^ = 36.5% (24 h) Ewazid^®^ Silgur F46 = 31% (24 h) Fossil Shied^®^ Istant White = 100% (24 h) Fossil Shield^®^ 90.0 White = 92.3% (24 h)	[112]	After 48 h, all the tested DE caused 100% of *D. gallinae* mortality
*Dermanyssus* *gallinae*	Dermanyssidae	Acarina	All stages	Diamol KMTSilicoSec^®^FS 90.0^®^ProtectIt	NA	NA	Dry	Diamol KMT = 60% (LT50 = 3 days) SilicoSec^®^ = 55% (LT50 = 3 days) Fossil Shield 90.0^®^ = 30% (LT50: 3 days) ProtectIt^®^ = 57% (LT50: 3 days)	[93]	The addition of the entomopathogenic fungi do not change the repellency of DEs
*Dermanyssus* *gallinae*	Dermanyssidae	Acarina	All stages	Diamol	NA	NA	Dry DE + *Beauveria bassiana*	Mortality 89.1% (H.R. 75%) Mortality 78.6% (H.R. 85%)	[94]	Synergistic interactions when applied simultaneously
*Dermanyssus* *gallinae*	Dermanyssidae	Acarina	Adult female	Diamol SilicoSec^®^	NA	NA	Dry	Low efficacy compared to a pure synthetic amorphous silica products	[130]	
*Menacanthus* *stramineus*	Menoponidae	Phthiraptera	All stages	Organic D/Earth^®^	NA	NA	DE mixed with sand (1:9)	60.4%–95.2%	[126]	
*Onythonyssus* *sylvarium*	Macronyssidae	Acari	All stages	NA	NA	NA	DE suspended in deionized water	Low efficacy, it reduced the mite population only if applied for 2 consecutive weeks.	[125]	
*Onythonyssus* *sylvarium*	Macronyssidae	Acari	All stages	Organic D/Earth^®^	NA	NA	DE mixed with sand (1:9)	29.1–97.5%	[126]	Data refer to control over 4 weeks of dust box use
*Onythonyssus* *sylvarium*	Macronyssidae	Acari	All stages	Food-grade DE	NA	NA	Food-Grade DE mixed with sand (1:9)	When dust boxes were used, the northern fowl mite populations on flocks grew slowly (<100 mites)	[127]	
*Protocalliphora* spp.	Calliphoridae	Diptera	All stages	Drione Crawling Insect Killer	NA	NA	Drione Crawling Insect Killer	Lower number of fleas in nests treated with DE	[129]	38.12% DE + 0.2% pyrethrins + 1.0% piperonyl butoxide
*Rhipicephalus* *microplus*	Ixodidae	Ixodida	All stages	Keep Dry^®^	86	NA	IP 46 + DE (pellets)	The combination effectively suppressed the population of *R. microplus* and reduced the female oviposition period	[121]	Pellets: Vermiculite (AgroFloc) + DE (KeepDry^®^) + SiO_2_

### 6.3. DEs to Control Crop Pests 

Although DEs are not widely used by farmers to control arthropod pests, several studies reported their efficacy to control pests of agricultural interest such as soft-bodied insects, ants, and moths [42,131,132,133,134] (Table 3). It is generally advisable to use DE as an adjuvant rather than an active ingredient alone, considering the wide range of environmental conditions during the application [135]. Indeed, studies about DE effectiveness toward crop-damaging arthropods mainly focused on their use in combination with other products such as essential oils or entomopathogenic fungi [42,92,132,136,137]. For instance, a study conducted on the green peach aphid, *Myzus persicae* (Sulzer) (Hemiptera: Aphididae), reported that a DE + the essential oil of *Thymus capitatus* (L.) (Lamiaceae) caused mortality higher than 95% through in vitro bioassays [136]. The adoption of DE in solid form or suspended in water with neem oil extracted from *Azadirachta indica* A. Juss. (Meliaceae), also protects maize and tomato plants, causing a decrease in the number of larvae of the southern armyworm, *Spodoptera eridania* Stoll (Lepidoptera: Noctuidae) and fall armyworm, *Spodoptera frugiperda* Smith & Abbot (Lepidoptera: Noctuidae) [132]. In addition, Fossil Shield^®^, already adopted to control the red poultry mite [130], was proved to increase the efficacy of neem oil extract against cowpea aphid, *Aphis craccivora* Koch (Hemiptera: Aphididae) on the yardlong beans, *Vigna unguiculata* subsp. *sesquipedalis* L. (Fabaceae) [138]. Evaluating side effects on the aphid predator *Menochilus sexmaculatus* F. (Coleoptera: Coccinellidae), the toxicity of DE + neem oil was lower than that of the recommended chemical insecticide [138]. The same mixture, i.e., Fossil Shield^®^ + neem oil, was evaluated against *M. persicae* (Sulzer) on globe artichoke *Cynara cardunculus* var. *scolymus* (L.) (Asteraceae) with promising results. The aphid population was reduced by 97% the day after the second spray [139]. Interestingly, the combination had a low impact on *M. persicae* common predators *Chrysoperla carnea* (Stephens) (Neuroptera: Chrysopidae), *Orius* spp. (Hemiptera: Antochoridae), *Coccinella* spp. (Coleoptera: Coccinellidae), and *Scymnus* spp. (Coleoptera: Coccinellidae), while *C. carnea* and *Orius* spp. were found to be more susceptible than the two coccinellids to that combination [139]. These results substantiated the findings by Ulrichs et al. [138], outlining the lower susceptibility of coccinellid predators to neem oil and DEs. In contact bioassays, DEs were also low risk toward predators of the spider mite, *Tetranychus urticae* Koch (Trombidiformes: Tetranychidae), such as *Phytoseiulus persimilis* Athias-Henriot (Mesostigmata: Phytoseiidae), *Neoseiulus fallacis* Garman (Mesostigmata: Phytoseiidae), and *Stethorus punctillum* (Weise) (Coleoptera: Coccinellidae), when DEs were tested in contact bioassay [140]. 

The synergistic interaction between DE and entomopathogenic fungi has been also evaluated toward an extremely wide range of agricultural pests [42,92]. For instance, a study on the western flower thrips, *Franklinella occidentalis* Pergande (Thysanoptera: Thripidae) highlighted that combining the entomopathogenic fungi, *Metarhizium flavoviride* (Gams and Rozsypal) (syn. *Metarhizium anisopliae* var. Acridum, pro parte) (Hyphomycetes: Deuteromycotina), with a commercial DE resulted in higher mortality of the thrips compared to the efficacies of each compound alone [92]. Synergistic interaction between DE and entomopathogenic fungi has been also reported for *T. infestans* [117], the silverleaf whitefly *Bemisia tabaci* (Gennadius) (Hemiptera: Aleyrodidae) [137], the cotton aphid, *Aphis gossypii* Glover (Hemiptera: Aphididae) [42], the indianmeal moth, *Plodia interpunctella* (Hübner), the almond moth, *Ephestia cautella* (Walker), and the Mediterranean flour moth, *Ephestia kuehniella* Zeller (Lepidoptera: Pyralidae) [141]. The insecticidal activity of DEs + fungi was evaluated toward the fire ant, *Solenopsis invicta* Buren (Hymenoptera: Formicidae) [131], which is responsible for a decline in production through direct predation on different plant parts (e.g. roots, fruits, flowers, stems), with reduction estimated to 15 to 33% in soybean, 20 to 35% in potato crops, or 50% in eggplant [141]. The effects of the combination of DE + *B. bassiana* toward healthy fire ants do not greatly increase the effect of *B. bassiana* alone, but testing DE alone in ants infected by *Thelohania solenopsae* (a common intracellular pathogen of fire ants) led to high insecticidal activity of DE, suggesting the synergistic interaction between *T. solenopsae* and DE [131].

## 7. DEs in Real-Scale Pest Management

The gradual withdrawal of active ingredients from the chemical-based pest management and the necessity to place insect control under the principles of IPM [142] led to the “re-evaluation” of inert dusts as a novel, effective, and sustainable management of arthropod pests and vectors all over the world. This is the main reason behind the recent popularity of DE formulations in pest management strategies [2], although commercial products (“Naaki” in Germany and “Neosyl” in England) have been available for stored-product protection since the 1930s [143]. In the following years, various enhanced or modified DE formulations have been created and evaluated, under extensive laboratory research, and some of them have reached the market as commercial formulations.

However, what are their potentials in praxis? Since several reports have questioned the compatibility of DEs with modern pest management programs, in this chapter, factors in terms of their potential impact in real scale applications (particularly as stored product protectants), including their utilization to reduce the standard application doses of residual insecticides and the role that DEs could have in resistance management will be discussed. Additional data of other relatively promising substances will be evaluated, aiming to expand the list of viable alternatives to hazardous chemicals that can be used as protectants in the food industry and beyond. 

Virtually all the “classic” papers on the insecticidal efficacy of DEs examine formulations at a laboratory scale, with scarce data to be available in the literature regarding applications in large-scale scenarios. DEs have been approved for arthropod pest control, and commercial formulations are currently available as effective grain protectants. However, the grain industry is reluctant to use them for direct mixture with grains, as DE particles can adversely affect some physical and mechanical properties of the treated grain, obstructing their wider use as grain protectants. Indeed, for a satisfactory level of efficacy, the commercially available DE formulations should be applied at doses between 400 and 1000 ppm, but even in this case, adverse effects cannot be avoided [2,30]. However, several reports suggest that using DEs at concentrations lower than those indicated on the label could cause a sufficient reduction in the bulk density (test weight) of the grain [1,10,12,15]. Bulk density refers to a grading factor extensively used by the industry to determine the grain price, and its reduction through DE applications is of major importance. Korunić [10] examined 42 DE dusts from around the world and found significant correlations between DE insecticidal efficacy and adherence to kernels with bulk density reduction. In a later report, Korunić et al. [144] stated that the insecticidal efficacy and bulk density reduction could be linked by the capacity of a given DE to adhere to surfaces, which, eventually, is positively correlated with the insecticidal value of a given DE. Furthermore, when the DE particles are attached to the surface of the kernels, the spaces among the kernels increase, affecting their flowability, especially in mechanized handling systems. Jackson and Webley [145] found that when 0.5 g/kg of DE was applied on maize, the flow rate was reduced by about 39%. Apart from the grain industry, the milling industry has also expressed concerns about using DE formulations, as the presence of DE particles in the grain can damage the milling machinery through abrasive action. To overcome these limitations, new ways of DE applications have been proposed, intending to make the most of their advantages.

**Table 3:** Local and commercial diatomaceous earths (DEs) examined against immature and adult stages of arthropod pests of agricultural interest. In addition to the mortality rates, the SiO_2_ content (%) and the diameter of particles (μm) are reported. NA = not available data.

Pest Species	Family	Order	Developmental Stage	Tested DE	SiO_2_ Content (%)	Ø Particles (µm)	Formulation	Mortality Rates	References	Notes
*Agrotis* *ipsilon*	Noctuidae	Lepidoptera	IV instar larvae	Local Raw DE	NA	NA	DE suspended in water	Low concentration: 10% High concentration: 70%	[146]	Low concentration 1 g/L High concentration 4 g/L
*Aphis* *craccivora*	Aphididae	Hemiptera	All stages	Fossil Shield^®^	73	5–30	DE suspended in water + neem oil	Mean number of aphids/plant 2.2	[138]	Limited effects on the predator *Menochilus sexmaculatus*
*Atta sexdens* *rubropilosa*	Formicidae	Hymenoptera	Ant colonies	NA	NA	NA	Dry	Inactivity of the nests: 5.26–31.57%	[147]	
*Bemisia* *argentifolii*	Aleurodidae	Hemiptera	Nymphs	HYFLO^®^	NA	NA	DE + *Isaria fumosorosea* (612 strain)	Infected/dead nymphs ranged between 53% and 42.8%, day 4 and 6 respectively	[137]	
*Epilachna* *vigintioctopunctata*	Coccinellidae	Coleoptera	Larvae + Adults	Fossil Shield 90.0 S^®^	60–80	5–30	DE	FS 90.0 Adult: ≈75%; Larvae: ≈40%	[148]	After 48 h
*Franklinella* *fusca*	Thripidae	Thysanoptera	All stages	Celite^®^610 (Deadzone)	85	NA	DE DE + Orthene^®^	% of thrips/plant DE < DE + Orthene^®^	[149]	Average number of thrips per plant 2 days after insecticide application
*Franklinella* *occidentalis*	Thripidae	Thysanoptera	Adults	Puliantagai^®^	85	NA	(i) DE + *M. flavoridae* (ii) DE + *M. flavoridae* + Imidacloprid	(i) LT_50_ 3.77 days (ii) LT_50_ 4.23 days	[92]	
*Franklinella* *occidentalis*	Thripidae	Thysanoptera	Nymphs	Puliantagai^®^	85	NA	(i) DE + *M. flavoridae* (ii) DE + *M. flavoridae* + Imidacloprid	(i) LT_50_ 4.26 days (ii) LT_50_ 2.45 days	[92]	
*Myzus* *persicae*	Aphididae	Hemiptera	All stages	NA	NA	NA	DE + *Thymus capitatus* EO	Mortality 97.84%	[136]	After 24 h
*Myzus* *persicae*	Aphididae	Hemiptera	All stages	Fossil Shield^®^	60–80	5–30	DE alone DE + neem oil	Fossil-Shield 68.6% Fossil-Shield + neem oil: 96.4%	[139]	Limited effects on *Chrysoperla carnea*, *Orius* spp., *Coccinella* spp., and *Scymnus* spp.
*Myzus* *persicae*	Aphididae	Hemiptera	All stages	Pyrisec^®^	NA	NA	DE DE + *Paecilomyces lilacinus*	DE: 35–40% after 8 days DE + *P. lilacinus*: 54.83% after 8 days	[42]	25% pyrethrum, 3.1% pipronylbutaoxide, and 97.5% diatomaceous earth (SilicoSec®)
*Myzus* *persicae*	Aphididae	Hemiptera	All stages	Pyrisec^®^	NA	NA	DE DE + *Paecilomyces lilacinus*	DE: ≈40% after 10 days DE + *P. lilacinus*: ≈60% after 10 days	[42]	25% pyrethrum, 3.1% pipronylbutaoxide, and 97.5% diatomaceous earth (SilicoSec®)
*Rhopalosiphum* *padi*	Aphididae	Hemiptera	All stages	NA	NA	2.6	DE suspended in water	Wheat plant dusted with different dosages of DE did not show any visible injury	[133]	Reduction in chlorophyll content was observed in them.
*Solenopsis* *invicta*	Formicidae	Hymenoptera	Worker ants	NA	NA	NA	Dry + fungi	*Thelohania solenopsae* + DE: 89% *Beauveria bassiana* + DE: <50%	[131]	After 10 days
*Spodoptera* *eridiana*	Noctuidae	Lepidoptera	II instar larvae	KeepDry^®^	86	15	DE suspended (alone) DE suspended + neem oil	DE: 46.6% DE + neem: 93.7%	[132]	DE highest dose non-additive synergistic
*Spodoptera* *exigua*	Noctuidae	Lepidoptera	III instar larvae	Sayan^®^	92	50	DE suspended in water	Mortality: 59.25%, concentration of 20%	[150]	After 72 h
*Spodoptera* *frugipreda*	Noctuidae	Lepidoptera	II instar larvae	Dezone	85	NA	DE suspended in water	High maize grain yield 7387 kg/ha.	[134]	
*Spodoptera* *frugiperda*	Noctuidae	Lepidoptera	II instar larvae	KeepDry^®^	86	15	DE suspended (alone) DE suspended + neem oil	DE: 76.2% DE + neem: 66.6%	[132]	DE highest dose additive effect
*Spodoptera* *littura*	Noctuidae	Lepidoptera	III instar larvae	Fossil Shield 90.0^®^	60–80	5–30	DE suspended in water	≈70% after 48 h	[148]	
*Tetranychus* *urticae*	Acarina	Tetranychidae	Adult (♀)	DE_cide	67	NA	DE suspended in water	Contact mortality: 24.6%	[140]	Limited impact on TSSM predators (*Phytoseiulus persimilis*, *Neoseiulus fallacis* and *Stethorus punctillum*).

## 8. DE Applications for Structural Treatments

DEs leave no harmful residues in the surfaces applied and hence, applications could be carried out in food and processing facilities. Korunić et al. [12] reported that the treatment of hard wheat with either 50 or 300 ppm of Protect-It^®^ had no significant effect on the milling, analytical, rheological, or baking quality, and these doses did not affect the properties for pasta production, while 100 to 900 ppm on barley showed no differences in malting quality characteristics. Desmarchelier and Dines [151] reported that treatments with Dryacide^®^ did not affect flour quality, as determined by the volume of sponge cakes and the production of carbon dioxide by fermenting dough. Aldryhim [152] found no evidence of an adverse effect on wheat seed germination, wheat flour, and baking quality, using Dryacide^®^.

Another advantageous feature of DEs is their persistence and stability in a wide range of temperatures [153,154], as compared with contact insecticides [155,156]. Arthur [39] exposed adults of *T. castaneum* and *T. confusum* to filter papers containing 0.5 mg/cm^2^ of Protect-It^®^ and reported a positive effect of temperature and exposure interval on insect mortality, along with a negative effect of humidity.

The utilization of DEs has been addressed as a good way to strengthen the effects of heat treatments, as the exposed insects are expected to die earlier due to increased desiccation. According to Fields et al. [157], the complete control of *T. confusum* in an oat mill could be achieved after treatment at 41 °C for 13–22 h, when DEs are combined with heat. In contrast, heat alone caused the same results after 32–38 h exposure at a sufficiently higher temperature, 47 °C [157]. Additional data by Dowdy [158] and Dowdy and Fields [159] indicated that DEs appear to be of value in areas where lethal temperatures cannot be reached during heat treatment applications. Moreover, even after the treatments combining heat with DEs, delayed mortality may occur after a while for the remaining insects due to the residual toxicity of DEs [159].

Laboratory studies have been conducted to evaluate the insecticidal efficacy of different DE formulations when applied directly in different types of surfaces, such as concrete, ceramic, plywood, plastic, metal, etc. [39,160,161,162,163,164]. In general, lower doses are required on some surfaces, such as metal and glass, compared with surfaces with a rougher construction, such as wood and concrete [160,163,164,165]. Collins and Cook [162] reported that 5 g/m^2^ of SilicoSec^®^ was just as effective as 20 g/m^2^ to achieve mortalities above 86% of different stored product insect and mite species after one week of exposure to glass and plastic surfaces. This observation is in accordance with the reports of Cook [166], Mewis and Ulrichs [37], and Athanassiou et al. [15], indicating that there is a limitation in the amount of DE particles the insects can pick up. In general, insects seem to pick up DE particles more easily if the formulation is equally applied onto the surface (e.g., on a Petri dish) and not adhered on the grain kernels [15]. In the latter case, DE particles are also likely to lose effectiveness by lipid absorption from the external part of the kernel [2].

Cleaning and sanitation before DE structural treatments is a key element in pest management practices since the presence of food may increase insect survival rates [159,167]. Arthur [39] using 0.5 mg/cm^2^ of Protect-It^®^ in plastic surface against *T. castaneum* and *T. confusum* emphasized the importance to eliminate the presence of food materials within the storage environment to maximize the effectiveness of the treatments. Dowdy [158] also addressed the impact of food in the effectiveness of treatments combining heat with some commercial DEs: Insecto^®^, Protect-It^®^, Concern^®^, and Natural Guard^®^ (VPG Co-op Gardening Group, Inc., Bonham, TX, USA). Access to food significantly decreased insect mortality, providing an average between 21 and 88% of individuals fed and not fed respectively, 7 d after the treatment. Similar results have been published using other inert dust formulations, as food may provide water and nutrition that can lead to increased and prolonged insect survival, which may allow the continuance of the infestation for a certain period and the concomitant progeny production [168,169,170].

Although most laboratory studies tended to prefer dry DE applications over the use of slurry solutions [15,28,153,161,171], the reverse is probably more desirable in commercial practice. Slurries may be used easier in their application by the personnel, as there is a need to avoid exposure to the very dusty atmospheres created by dry-blown methods [153,165].

## 9. Other Relative Promising Substances

A plethora of other inert dusts has been also tested for their toxicity against arthropod species, with special reference to stored product pests. In general, inert dusts/materials can be categorized according to their chemical composition or level of activity in four wide groups: (*a*) clays, sand, kaolin, paddy husk ash, wood, and volcanic ash, (*b*) katelsous (rock phosphate and ground sulfur), lime (calcium hydroxide), limestone (calcium carbonate), and salt (sodium chloride), (*c*) synthetic silica aerogels produced by drying aqueous solutions of sodium silicate and (*d*) dusts containing natural silica, including DEs [1,2,172,173]. Zeolites (alkali metal aluminum silicates) have been also included in this group by Subramanyam and Roesli [2], since these substances have similar physical properties with DEs. In addition, Golob [173] divided the DE formulations into two groups, addressing the modified DE formulations that contain over 98% silicon dioxide (compared to the 90% silicon dioxide of the natural dusts) as the fifth group of inert dusts. 

Zeolites are among the most promising alternatives to DEs, and their potentials in food and agriculture are well described by Eroglu et al. [5]. Nevertheless, regarding stored product protection, there are disproportionally few data as compared with DEs, although the interest for zeolites in stored product protection has been increased [174,175,176,177,178]. Zeolites’ particle size effect, adherence to kernels, and influence on the test weight of grains have been examined by Rumbos et al. [177], showing similar trends with DEs. The results of these studies encourage further research to evaluate the use of zeolites as grain protectants but also to surface treatments or “crack and crevice” applications. Attempts have been also made to evaluate the insecticidal efficacy of other inert dusts, to use them in modern pest management at the post-harvest stages of durable agricultural commodities [2]. Even some of the currently existing DEs cannot be considered as pure DE formulations, as they contain additional inert materials that have a certain insecticidal action and can be drastically modified to obtain increased efficacy [11].

## 10. Conclusions and Future Challenges

The need to gradually withdraw from the chemical-based pesticide policies to more sustainable and ecological approaches is, at the present, one of the most challenging aspects of pest management. The current decrease in the registered pesticides will undoubtedly continue, increasing simultaneously the need to develop novel, effective but also ecologically compatible substances. On the other hand, the introduction of a new pesticide is a costly and long process, making the total overdrawn from the traditional protectants an unrealistic scenario. Thus, inert dusts such as DEs might have an important role to play in future pest management strategies, ensuring an abundant supply of safe and healthy food and feed. DEs hold great potential as carriers of common insecticides, minimizing the required application doses of the latter. In addition to synergistic effects, combined applications may also alleviate the negative effects of the substances and can be more compatible with the desired criteria for food safety and protection of human health and the environment. However, the introduction of other agents must be always appraised under the prism of the potential negative effects they may hold, such as for instance the adoption of entomopathogenic fungus agents [179]. Nevertheless, the application of insecticides and acaricides with a different mode of action may be a solution for the control of resistant arthropod populations, which is a hot topic in modern Integrated Pest Management [180,181,182]. Although there are plenty of data for their insecticidal and acaricidal properties, little progress has been made regarding the optimal processing of DE dusts used as insecticides and acaricides. Today, most commercial DE formulations are prepared through a basic process of quarrying, drying, and milling the mined heterogeneous rocks. This simplistic treatment leads eventually to formulations with great variability in their physicochemical characteristics, influencing simultaneously their insecticidal/acaricidal properties but also some properties of the commodity itself. Therefore, more specific methods of processing must be found to standardize the production of dusts bearing the most desirable features for increased insecticidal efficacy. 

Application methods and systems of DEs are also an issue of major importance, requiring additional investigation. Even with the current DE formulations, different application techniques, such as using slurries or treating only partial layers of the food, should be explored. Thus, research should be conducted under a range of food-handling establishments to design effective protocols for pest management but also to determine the effects of sanitation on the performance of DE dusts. Such real-scale applications may highlight the potential of DEs and explore ways of integrating DE applications within the total pest/vector management program in food industry, agricultural and urban settings.

The data from laboratory studies underline the insecticidal and acaricidal value of DEs under a wide range of arthropods. Further analysis must be conducted toward this direction not only to identify all the target species but also to investigate the overall outcome of DEs in non-target species. Indeed, by examining the current literature, we observed that non-target effects of DE have been evaluated only on a limited number of natural enemies of crop pests, with special reference to aphidophagous coccinellids, lacewings, anthochorids, and Phytoseiidae mites, showing limited consequences for these important biocontrol agents. In this promising scenario, further research should be devoted in understanding the potential non-target effects of DE-based formulations.

## References

[1] Korunić Z. (1998). Diatomaceous earths, a group of natural insecticides. J. Stored Prod. Res..

[2] Subramanyam B., Roesli R., Subramanyam B., Hagstrum D.W. (2000). Inert Dusts. Alternatives to Pesticides in Stored-Product IPM.

[3] Fernandez M.A., Bellotti N. (2017). Silica-based bioactive solids obtained from modified diatomaceous earths to be used as antimicrobial filler material. Mater. Lett..

[4] Bellotti N., Deyá C. (2019). Chapter 14. Natural products applied to antimicrobial coatings. Stud. Nat. Prod. Chem..

[5] Eroglu N., Emekci M., Athanassiou C.G. (2017). Applications of natural zeolites on agriculture and food production. J. Sci. Food Agric..

[6] Arthur F.H., Puterka G.J. (2002). Evaluation of kaolinite-based particle films to control *Tribolium* species (Coleoptera: Tenebrionidae). J. Stored Prod. Res..

[7] Athanassiou C.G., Kavallieratos N.G., Benelli G., Losić D., Usha Rani P., Desneux N. (2018). Nanoparticles for pest control: Current status and future perspectives. J. Pest Sci..

[8] Korunić Z. (2016). Overview of undesirable effects of using diatomaceous earths for direct mixing with grains. Pestic. Fitomed..

[9] Vayias B.J., Athanassiou C.G., Korunić Z., Rozman V. (2009). Evaluation of natural diatomaceous earth deposits from south-eastern Europe for stored-grain protection: The effect of particle size. Pest Manag. Sci..

[10] Korunić Z. (1997). Rapid assessment of the insecticidal value of diatomaceous earths without conducting bioassays. J. Stored Prod. Res..

[11] Baliota G.V., Athanassiou C.G. (2020). Evaluation of a Greek diatomaceous earth for stored product insect control and techniques that maximize its insecticidal efficacy. Appl. Sci..

[12] Korunić Z., Fields P.G., Kovacs M.I.P., Noll J.S., Lukow O.M., Demianyk C.J., Shibley K.J. (1996). The effect of diatomaceous earth on grain quality. Postharvest Biol. Technol..

[13] Kavallieratos N.G., Athanassiou C.G., Paschalidou F.G., Andris N.S., Tomanovic Z. (2005). Influence of grain type on the insecticidal efficacy of two diatomaceous earth formulations against *Rhyzopertha dominica* (F.) (Coleoptera: Bostrychidae). Pest Manag. Sci..

[14] Athanassiou C.G., Kavallieratos N.G. (2005). Insecticidal effect, and adherence of PyriSec^®^ in different grain commodities. Crop Prot..

[15] Athanassiou C.G., Kavallieratos N.G., Vayias B.J., Tomanović Z., Petrović A., Rozman V., Adler C., Korunić Z., Milovanović D. (2011). Laboratory evaluation of diatomaceous earth deposits mined from several locations in central and southeastern Europe as potential protectants against coleopteran grain pests. Crop Prot..

[16] Rigaux M., Haubruge E., Fields P.G. (2001). Mechanisms for tolerance to diatomaceous earth between strains of *Tribolium castaneum* (Coleoptera: Tenebrionidae). Entomol. Exp. Appl..

[17] Vayias B.J., Athanassiou C.G., Kavallieratos N.G., Buchelos C.T. (2006). Susceptibility of different European populations of *Tribolium confusum* (Coleoptera: Tenebrionidae) to five diatomaceous earth formulations. J. Econ. Entomol..

[18] Vayias B.J., Athanassiou C.G., Buchelos C.T. (2008). Evaluation of resistance development by *Tribolium confusum* du Val (Coleoptera: Tenebrionidae) to diatomaceous earth under laboratory selection. J. Stored Prod. Res..

[19] Athanassiou C.G., Kavallieratos N.G., Andris N.S. (2004). Insecticidal effect of three diatomaceous earth formulations against adults of *Sitophilus oryzae* (Coleoptera: Curculionidae) and *Tribolium confusum* (Coleoptera: Tenebrionidae) on oat, rye, and triticale. J. Econ. Entomol..

[20] Athanassiou C.G., Vayias B.J., Dimizas C.B., Kavallieratos N.G., Papagregoriou A.S., Buchelos C.T. (2005). Insecticidal efficacy of diatomaceous earth against *Sitophilus oryzae* (L.) (Coleoptera: Curculionidae) and *Tribolium confusum* Du Val (Coleoptera: Tenebrionidae) on stored wheat: Influence of dose rate, temperature and exposure interval. J. Stored Prod. Res..

[21] Nayak M.K., Daglish G.J., Athanassiou C.G., Arthur F.H. (2018). Importance of Stored Product Insects. Recent Advances in Stored Product Protection.

[22] Campbell J.F., Arthur F.H., Mullen M.A. (2004). Insect management in food processing facilities. Adv. Food Nutr. Res..

[23] Stejskal V., Hubert J., Aulocky R., Kucerova Z. (2015). Overview of present and past and pest-associated risks in stored food and feed products: European perspective. J. Stored Prod. Res..

[24] Hagstrum D.W., Athanassiou C.G. (2019). Improving stored product insect management: From theory to practice. Insects.

[25] Vayias B.J., Athanassiou C.G. (2004). Factors affecting effcacy of the diatomaceous earth formulation SilicoSec against adults and larvae of the confused beetle *Tribolium confusum* Du Val (Coleoptera: Tenebrionidae). Crop Prot..

[26] Pixton S.W. (1967). Moisture content-its significance and measurement in stored products. J. Stored Prod. Res..

[27] Pixton S.W., Warburton S. (1971). Moisture content/relative humidity equilibrium of some cereal grains at different temperatures. J. Stored Prod. Res..

[28] Athanassiou C.G., Korunić Z. (2007). Evaluation of two new diatomaceous earth formulations, enhanced with abamectin and bitterbarkomycin, against four stored-grain beetle species. J. Stored Prod. Res..

[29] Athanassiou C.G., Arthur F.H., Athanassiou C.G., Arthur F.H. (2018). Bacterial Insecticides and Inert Materials. Recent Advances in Stored Product Protection.

[30] Fields P., Korunić Z. (2000). The effect of grain moisture content and temperature on the efficacy of diatomaceous earths from different geographical locations against stored product beetles. J. Stored Prod. Res..

[31] Athanassiou C.G., Kavallieratos N.G., Tsakiri J.B., Xyrafidis S.N., Vayias B.J. (2006). Effect of temperature and humidity on insecticidal effect of SilicoSec against *Ephestia kuehniella* (Lepidoptera: Pyralidae) larvae. J. Econ. Entomol..

[32] Aldryhim Y.N. (1993). Combination of classes of wheat and environmental factors affecting the efficacy of amorphous silica dust, dryacide, against *Rhyzopertha dominica* (F.). J. Stored Prod. Res..

[33] Palyvos N.E., Athanassiou C.G., Kavallieratos N.G. (2006). Acaricidal effect of a diatomaceous earth formulation against *Tyrophagus putrescentiae* (Astigmata: Acaridae) and its predator *Cheyletus malaccensis* (Prostigmata: Cheyletidae) in four grain commodities. J. Econ. Entomol..

[34] Athanassiou C.G., Korunić Z., Vayias B.J. (2009). Diatomaceous earths enhance the insecticidal effect of bitterbarkomycin against stored-grain insects. Crop Prot..

[35] Rudolph D. (1982). Occurrence, properties and biological implications of the active uptake of water vapour from the atmosphere in Psocoptera. J. Insect Physiol..

[36] Rudolph D. (1982). Site, process and mechanism of active uptake of water vapour from the atmosphere in the Psocoptera. J. Insect Physiol..

[37] Mewis I., Ulrichs C. (2001). Action of amorphous diatomaceous earth against different stages of the stored product pests *Tribolium confusum* (Coleoptera: Tenebrionidae), *Tenebrio molitor* (Coleoptera: Tenebrionidae), *Sitophilus granarius* (Coleoptera: Curculionidae) and *Plodia interpunctella* (Lepidoptera: Pyralidae). J. Stored Prod. Res..

[38] Arthur F.H. (2000). Toxicity of diatomaceous earth to red flour beetles and confused flour beetles (Coleoptera: Tenebrionidae): Effects of temperature and relative humidity. J. Econ. Entomol..

[39] Arthur F.H. (2000). Impact of food source on survival of red flour beetles and confused flour beetles (Coleoptera: Tenebrionidae) exposed to diatomaceous earth. J. Econ. Entomol..

[40] Athanassiou C.G., Kavallieratos N.G., Vayias J.B., Tsakiri J.B., Mikeli N.H., Meletsis C.M. (2008). Tomanović, Ž. Persistence and efficacy of *Metarhizium anisopliae* (Metschnikoff) Sorokin (Deuteromycotina: Hyphomycetes) and diatomaceous earth against *Sitophilus oryzae* (L.) (Coleoptera: Curculionidae) and *Rhyzopertha dominica* (F.) (Coleoptera: Bostrychidae) on wheat and maize. Crop Prot..

[41] Wakil W., Riasat T., Lord J.C. (2013). Effects of combined thiamethoxam and diatomaceous earth on mortality and progeny production of four Pakistani populations of *Rhyzopertha dominica* (Coleoptera: Bostrichidae) on wheat, rice and maize. J. Stored Prod. Res..

[42] Wakil W., Schmitt T., Kavallieratos N.G. (2021). Performance of diatomaceous earth and imidacloprid as wheat, rice and maize protectants against four stored-grain insect pests. J Stored Prod Res.

[43] Ceruti F.C., Lazzari S.M.N. (2005). Combination of diatomaceous earth and powder deltamethrin for insect control in stored corn. Rev. Bras. Entomol..

[44] Arthur F.H. (2004). Evaluation of a new insecticide formulation (F2) as a protectant of stored wheat, maize, and rice. J. Stored Prod. Res..

[45] Awais M., Mansoor-ul-Hasan, Sagheer M., Asif M.U., Ali Q., Zaman S. (2019). Efficacy of diatomaceous earth and insect growth regulators against *Tribolium castaneum* (Herbst) (Coleoptera: Tenebrionidae). Sci. Lett..

[46] Awais M., Zeeshan M., Mansoor-ul-Hasan, Sagheer M., Asif M.U., Ali Q., Zaman S. (2020). Combined effect of diatomaceous earth and two insect growth regulators against *Trogoderma granarium* (Coleoptera: Dermestidae). Sci. Lett..

[47] Arthur F.H. (2004). Evaluation of methoprene alone and in combination with diatomaceous earth to control *Rhyzopertha dominica* (Coleoptera: Bostrichidae) on stored wheat. J. Stored Prod. Res..

[48] Athanassiou C.G. (2006). Toxicity of beta cyfluthrin applied alone or in combination with diatomaceous earth against adults of *Sitophilus oryzae* (L.) (Coleoptera: Curculionidae) and *Tribolium confusum* DuVal (Coleoptera: Tenebrionidae) on stored wheat. Crop Prot..

[49] Wakil W., Riasat T., Ashfaq M. (2012). Residual efficacy of thiamethoxam, *Beauveria bassiana* (Balsamo) Vuillemin, and diatomaceous earth formulation against *Rhyzopertha dominica* F. (Coleoptera: Bostrychidae). J. Pest Sci..

[50] Korunić Z., Kalinovic I., Liska A., Hamel D., Carvalho M.O., Fields P.G., Adler C.S., Arthur F.H., Athanassiou C.G., Campbell J.F., Fleurat-Lessard F., Flinn P.W., Hodges R.J., Isikber A.A. (2010). Long Term Effectiveness of the Mixture of Diatomaceous Earth and Deltamethrin on Wheat. Proceedings of the Tenth International Working Conference on Stored Product Protection.

[51] Wakil W., Schmitt T. (2015). Field trials on the efficacy of *Beauveria bassiana*, diatomaceous earth and Imidacloprid for the protection of wheat grains from four major stored grain insect pests. J. Stored Prod. Res..

[52] Dhuyo A.R., Ahmad S. (2007). Evaluation of fungus *Beauveria bassiana* (Bals.) infectivity to the larger grain borer *Prostephanus truncatus* (Horn). Pak. Entomol..

[53] Lord J.C. (2009). Efficacy of *Beauveria bassiana* for control of *Tribolium castaneum* with reduced oxygen and increased carbon dioxide. J. Appl. Entomol..

[54] Phillips T.W., Throne J.E. (2010). Biorational approaches to managing stored-product insects. Annu. Rev. Entomol..

[55] Ahmed B.I. (2010). Potentials of entomopathogenic fungi in controlling the menace of maize weevil *Sitophilus zeamais* Motsch (Coleoptera: Curculionidae) on stored maize grain. Arch. Phytopathol. Pflanzenschutz.

[56] Kavallieratos N.G., Athanassiou C.G., Aountala M.M., Kontodimas D.C. (2014). Evaluation of the entomopathogenic fungi *Beauveria bassiana*, *Metarhizium anisopliae*, and *Isaria fumosorosea* for control of *Sitophilus oryzae*. J. Food Prot..

[57] Batta Y.A. (2016). Recent advances in formulation and application of entomopathogenic fungi for biocontrol of stored-grain insects. Biocontrol Sci. Technol..

[58] Moore D., Lord J.C., Smith S.M., Subramanyam B., Hagstrum D.W. (2000). Pathogens. Alternatives to Pesticides in Stored-Product IPM.

[59] Lord J.C. (2005). Low humidity, moderate temperature and desiccant duct favor the efficacy of *Beauveria bassiana* (Hyphomycetes: Moniliales) for the lesser grain borer *Rhyzopertha dominica* (Coleoptera: Bostrychidae). Biol. Control.

[60] Akbar W., Lord J.C., Nechols J.R., Howard R.W. (2004). Diatomaceous earth increases the efficacy of *Beauveria bassiana* against *Tribolium castaneum* larvae and increases conidia attachment. J. Econ. Entomol..

[61] Michalaki M.P., Athanassiou C.G., Kavallieratos N.G., Batta Y.A., Balotis G.N. (2006). Effectiveness of *Metarhizium anisopliae* (MetschinkoV) Sorokin applied alone or in combination with diatomaceous earth against *Tribolium confusum* Du Val larvae: Influence of temperature, relative humidity and type of commodity. Crop Prot..

[62] Vassilakos T.N., Athanassiou C.G., Kavallieratos N.G., Vayias B.J. (2006). Influence of temperature on the insecticidal effect of *Beauveria bassiana* in combination with diatomaceous earth against *Rhyzopertha dominica* and *Sitophilus oryzae* on stored wheat. Biol. Control.

[63] Hansen L.S., Steenberg T. (2007). Combining larval parasitoids and an entomopathogenic fungus for biological control of *Sitophilus granarius* (Coleoptera: Curculionidae) in stored grain. Biol. Control.

[64] Wakil W., Ghazanfar M.U. (2010). Entomopathogenic fungus as a biological control agent against *Rhyzopertha dominica* F. (Coleoptera: Bostrychidae) on stored wheat. Arch. Phytopathol. Pflanzenschutz.

[65] Lord J.C. (2001). Desiccant dusts synergize the effect of *Beauveria bassiana* (Hyphomycetes: Moniliales) on stored-grain beetles. J. Econ. Entomol..

[66] Moore D., Douro-Kpindou O.K., Jenkins N.E., Lomer C.J. (1996). Effects of moisture content and temperature on storage of *Metarhizium flavoviride* conidia. Biocontrol Sci. Technol..

[67] Sheeba G., Seshardi S., Raja N., Janarthanan S., Ignacinutha S. (2001). Efficacy of *Beauveria bassiana* for control of the rice weevil *Sitophilus oryzae* (L.) (Coleoptera: Curculionidae). Appl. Entomol. Zool..

[68] Batta Y.A. (2005). Control of the lesser grain borer (*Rhyzopertha dominica* Fab., Coleoptera: Bostrichidae) by treatments with residual formulations of *Metarhizium anisopliae* (Metchnikoff) Sorokin (Deuteromycotina: Hyphomycetes). J. Stored Prod. Res..

[69] Kavallieratos N.G., Athanassiou C.G., Michalaki M.P., Batta Y.A., Rigatos H.A., Pashalidou F.G., Balotis G.N., Tomanović Ž., Vayias B.J. (2006). Effect of the combined use of *Metarhizium anisopliae* (Metschinkoff) Sorokin and diatomaceous earth for the control of three stored-product beetle species. Crop Prot..

[70] Athanassiou C.G., Steenberg T. (2007). Insecticidal effect of *Beauveria bassiana* (Balsamo) Vuillemin (Ascomycota: Hypocreaes) in combination with three diatomaceous earth formulations against *Sitophilus granarius* (L.) (Coleoptera: Curculionidae). Biol. Control.

[71] Batta Y.A. (2008). Control of main stored-grain insects with new formulations of entomopathogenic fungi in diatomaceous earth dusts. Int. J. Food Eng..

[72] Riasat T., Wakil W., Ashfaq M., Sahi S.T. (2011). Effect of *Beauveria bassiana* mixed with diatomaceous earth on mortality, mycosis and sporulation of *Rhyzopertha dominica* on stored wheat. Phytoparasitica.

[73] Wakil W., Riasat T., Ghazanfar M.U., Kwon Y.J., Shaheen F.A. (2011). Aptness of *Beauveria bassiana* and enhanced diatomaceous earth (DEBBM) for control of *Rhyzopertha dominica* F. Entomol. Res..

[74] Dal Bello G., Fusé C., Juarez P., Pedrini N., Imaz A., Padin S. (2011). Insecticidal effect of fenitrothion, diatomaceous earth and *Beauveria bassiana* against Coleopteran pests on stored grain. Integr. Prot. Stored Prod. IOBC/WPRS Bull..

[75] Sedehi A., Sedaghatfar E., Modarres-Najafabadi S.S. (2014). Studies on effect of the *Beauveria bassiana* on eggs and larvae of *Plodia interpunctella*. Can. J. Basic Appl. Sci..

[76] Shafighi Y., Ziaee M., Ghosta Y. (2014). Diatomaceous earth used against insect pests, applied alone or in combination with *Metarhizium anisopliae* and *Beauveria bassiana*. J. Plant Prot. Res..

[77] Rizwan M., Atta B., Rizwan M., Sabir A.M., Shah Z.U., Hussain M. (2019). Effect of the entomopathogenic fungus, *Beauveria bassiana*, combined with diatomaceous earth on the red flour beetle, *Tribolium castaneum* (Herbst) (Tenebrionidae: Coleoptera). Egypt J Biol Pest Control.

[78] Michalaki M.P., Athanassiou C.G., Steenberg T., Buchelos C.T. (2007). Effect of *Paecilomyces fumosoroseus* (Wise) Brown and Smith (Ascomycota: Hypocerales) alone or in combination with diatomaceous earth against *Tribolium confusum* Jacquelin du Val (Coleoptera: Tenebrionidae) and *Ephestia kuehniella* Zeller (Lepidoptera: Pyralidae). Biol. Control.

[79] Sabbour M.M., Abd-El-Aziz S.E., Sherief M.A. (2012). Efficacy of three entomopathogenic fungi alone or in combination with diatomaceous earth modifications for the control of three pyralid moths in stored grains. J. Plant Prot. Res..

[80] Riasat T., Wakil W., Yasin M., Kwon Y.J. (2013). Mixing of *Isaria fumosorosea* with enhanced diatomaceous earth and bitterbarkomycin for control of *Rhyzopertha dominica*. Entomol. Res..

[81] Sabbour M.M., Abd-El-Aziz S.E. (2014). Control of *Bruchidius incarnatus* and *Rhyzopertha dominica* using two entomopathogenic fungi alone or in combination with modified diatomaceous earth. Elixir Int. J. Ent..

[82] Athanassiou C.G., Rani P.U., Kavallieratos N.G., Singh D. (2014). The Use of Plant Extracts for Stored Product Protection. Advances in Plant Biopesticides.

[83] Campolo O., Giunti G., Russo A., Palmeri V., Zappalà L. (2018). Essential Oils in Stored Product Insect Pest Control. J. Food Qual..

[84] Fisher M.H., Mrozik H., Campbell W.C. (1989). Chemistry. Ivermectin and Abamectin.

[85] Yang F., Liang G., Xu Y., Lu Y., Zeng L. (2010). Diatomaceous earth enhances the toxicity of garlic, *Allium sativum*, essential oil against stored-product pests. J. Stored Prod. Res..

[86] Ziaee M., Moharramipour S., Francikowski J. (2014). The synergistic effects of *Carum copticum* essential oil on diatomaceous earth against *Sitophilus granarius* and *Tribolium confusum*. J. Asia Pac. Entomol..

[87] Korunić Z., Fields P.G. (2020). Evaluation of three new insecticide formulations based on inert dusts and botanicals against four stored-grain beetles. J. Stored Prod. Res..

[88] Campolo O., Romeo F.V., Malacrinò A., Laudani F., Carpinteri G., Fabroni S., Rapisarda P., Palmeri V. (2014). Effects of inert dusts applied alone and in combination with sweet orange essential oil against *Rhyzopertha dominica* (Coleoptera: Bostrichidae) and wheat microbial population. Ind. Crops Prod..

[89] Paponja I., Rozman V., Liška A. (2020). Natural formulation based on diatomaceous earth and botanicals against stored product insects. Insects.

[90] Akhtar Y., Isman M.B. (2016). Efficacy of diatomaceous earth and a DE-aerosol formulation against the common bed bug, *Cimex lectularius* Linnaeus in the laboratory. J. Pest Sci..

[91] Alkan M., Atay T., Ertürk S., Kepenekçi I. (2019). Comparison of bioactivities of native diatomaceous earth against Turkestan cockroach [*Blatta Lateralis* Walker (Blattodea: Blattidae)] Nymphs. Appl. Ecol. Environ. Res..

[92] Ge W., Du G., Zhang L., Li Z., Xiao G., Chen B. (2020). The Time–Concentration–Mortality Responses of Western Flower Thrips, *Frankliniella occidentalis*, to the Synergistic Interaction of Entomopathogenic Fungus *Metarhizium flavoviride*, Insecticides, and Diatomaceous Earth. Insects.

[93] Kilpinen O., Steenberg T. (2016). Repellent activity of desiccant dusts and conidia of the entomopathogenic fungus *Beauveria bassiana* when tested against poultry red mites (*Dermanyssus gallinae*) in laboratory experiments. Exp. Appl. Acarol..

[94] Steenberg T., Kilpinen O. (2014). Synergistic interaction between the fungus *Beauveria bassiana* and desiccant dusts applied against poultry red mites (*Dermanyssus gallinae*). Exp. Appl. Acarol..

[95] Akhoundi M., Bruel C., Izri A. (2019). A Harmful Effects of Bed Bug-Killing Method of Diatomaceous Earth on Human Health. J. Insect Sci..

[96] Hosseini S.A., Bazrafkan S., Vatandoost H., Abaei M.R., Ahmadi M.S., Tavassoli M., Shayeghi M. (2014). The insecticidal effect of diatomaceous earth against adults and nymphs of *Blattella germanica*. Asian Pac. J. Trop. Biomed..

[97] Di Giovanni F., Wilke A.B.B., Beier J.C., Pombi M., Mendoza-Roldan J.A., Desneux N., Canale A., Lucchi A., Dantas-Torres F., Otranto D. (2021). Parasitic strategies of arthropods of medical and veterinary importance. Entomol. Gen..

[98] Pan X., Wang X., Zhang F. (2020). New Insights into Cockroach Control: Using Functional Diversity of *Blattella germanica* Symbionts. Insects.

[99] Faulde M.K., Scharninghausen J.J., Cavaljuga S. (2006). Toxic and behavioural effects of different modified diatomaceous earths on the German cockroach, *Blattella germanica* (L.) (Orthoptera: Blattellidae) under simulated field conditions. J. Stored Prod. Res..

[100] Faulde M.K., Tisch M., Scharninghausen J.J. (2006). Efficacy of modified diatomaceous earth on different cockroach species (Orthoptera, Blattellidae) and silverfish (Thysanura, Lepismatidae). J. Pest Sci..

[101] Gao Y., Yu S., Li J., Sun P., Xiong M., Lei C., Zhang Z., Huang Q. (2018). Bioactivity of diatomaceous earth against the subterranean termite *Reticulitermes chinensis* Snyder (Isoptera: Rhinotermitidae). Environ. Sci. Pollut. Res..

[102] Özcan K., Tunaz H., Işikber A.A., Kubilay E.M. Lethal Effect of Turkısh Dıatomaceous Earth (Bgn-1) agaınst Adults of German Cockroaches (*Blatella Germanıca* L.). Contact Pesticides, Residual Products, and Plant Extracts. In Proceedings of the 12th International Working Conference on Stored Product Protection (IWCSPP).

[103] Ahmed S., Hassan B., Farooq M.U. (2018). Effect of biofertilizers and diatomaceous earth on life and movement of subterranean termites under laboratory conditions. Int. J. Trop. Insect Sci..

[104] Grace J.K., Yamamoto R.T. (1993). Diatomaceous earth is not a barrier to Formosan subterranean termites (Isoptera: Rhinotermitidae). Sociobiology.

[105] Lucky A. (2009). Urban Ants of North America and Europe: Identification, Biology, and Management. Syst. Entomol..

[106] Van Den Noortgate H., Sree S.P., Ostyn N., Lagrain B., Roefaers M., Wenseleers T., Martens J.A. (2018). Material properties determining insecticidal activity of activated carbon on the pharaoh ant (*Monomorium pharaonis*). J. Pest Sci..

[107] Al N., Tunaz H., Işikber A.A., Er M.K. Lethal Effect Of Turkish Diatomaceous Earth (K14) Against Adults Of American Cockroaches (Periplaneta americana L.). Proceedings of the International Biological, Agricultural And Life Science Congress.

[108] Akhtar Y., Isman M.B. (2013). Horizontal Transfer of Diatomaceous Earth and Botanical Insecticides in the Common Bed Bug, *Cimex lectularius* L.; Hemiptera: Cimicidae. PLoS ONE.

[109] Rodrigues J., Borges P.R., Fernandes É.K.K., Luz C. (2019). Activity of additives and their effect in formulations of *Metarhizium anisopliae* s.l. IP 46 against *Aedes aegypti* adults and on post mortem conidiogenesis. Acta Trop.

[110] Alves L.F.A., Oliveira D.G.P., Kasburg C.R., Nardelli M.S. (2019). Acaricidal Activity Of Inert Powders Against The Poultry Red Mite *Dermanyssus gallinae* (De Geer, 1778) (Mesostigmata: Dermanyssidae). Arch. Vet. Sci..

[111] Alves L.F.A., de Oliveira D.G.P., Pares R.B., Sparagano O.A.E., Godinho R.P. (2020). Association of mechanical cleaning and a liquid preparation of diatomaceous earth in the management of poultry red mite, *Dermanyssus gallinae* (Mesostigmata: Dermanyssidae). Exp. Appl. Acarol..

[112] Ulrichs C., Han Y.J., Abdelhamid M.T., Mewis I. (2020). Management of the poultry red mite, *Dermanyssus gallinae*, using silica-based acaricides. Exp. Appl. Acarol..

[113] Lilly D.G., Webb C.E., Doggett S.L. (2016). Evidence of Tolerance to Silica-Based Desiccant Dusts in a Pyrethroid-Resistant Strain of *Cimex lectularius* (Hemiptera: Cimicidae). Insects.

[114] Singh N., Wang C., Wang D., Cooper R., Zha C. (2016). Comparative Efficacy of Selected Dust Insecticides for Controlling *Cimex lectularius* (Hemiptera: Cimicidae). J. Econ. Entomol..

[115] Benoit J.B., Phillips S.A., Croxall T.J., Christensen B.S., Yoder J.A., Denlinger D.L. (2009). Addition of alarm pheromone components improves the effectiveness of desiccant dusts against *Cimex lectularius*. J. Med. Entomol..

[116] Pedrini N., Mijailovsky S.J., Girotti J.R., Stariolo R., Cardozo R.M., Gentile A., Juárez M.P. (2009). Control of pyrethroid-resistant chagas disease vectors with entomopathogenic fungi. PLoS Negl. Trop. Dis..

[117] Luz C., Rodrigues J., Rocha L.F.N. (2012). Diatomaceous earth and oil enhance effectiveness of *Metarhizium anisopliae* against *Triatoma infestans*. Acta Trop..

[118] Athanassiou C.G., Palyvos N.E. (2006). Laboratory evaluation of two diatomaceous earth formulations against *Blattisocius keegani* fox (Mesostigmata, Ascidae) and *Cheyletus malaccensis* Oudemans (Prostigmata, Cheyletidae). Biol. Control.

[119] Rodrigues J., Lobo L.S., Fernandes E.K.K., Luz C. (2015). Effect of formulated *Metarhizium anisopliae* on eggs and eclosing nymphs of *Triatoma infestans*. J. Appl. Entomol..

[120] Forlani L., Pedrini N., Juarez M.P. (2011). Contribution of the horizontal transmission of the entomopathogenic fungus *Beauveria bassiana* to the overall performance of a fungal powder formulation against *Triatoma infestans*. Res. Rep. Trop. Med..

[121] Santos T.R., da Paixão F.R.S., Catão A.M.L., Muniz E.R., Ribeiro-Silva C.S., Taveira S.F., Luz C., Mascarin G.M., Fernandes E.K.K., Marreto R.N. (2021). Inorganic pellets containing microsclerotia of *Metarhizium anisopliae*: A new technological platform for the biological control of the cattle tick *Rhipicephalus microplus*. Appl. Microbiol. Biotechnol..

[122] Kilpinen O., Roepstorff A., Permin A., Nørgaard-Nielsen G., Lawson L.G., Simonsen H.B. (2005). Influence of *Dermanyssus gallinae* and *Ascaridia galli* infections on behaviour and health of laying hens (*Gallus gallus domesticus*). Br. Poult. Sci..

[123] Chu T.T.H., Murano T., Uno Y., Usui T., Yamaguchi T. (2014). Molecular Detection of Avian Pathogens in Poultry Red Mite (Dermanyssus gallinae) Collected in Chicken Farms. J. Vet. Med. Sci..

[124] Sparagano O.A.E., Ho J. (2020). Parasitic Mite Fauna in Asian Poultry Farming Systems. Front. Vet. Sci..

[125] Mullens B.A., Soto D., Martin C.D., Callaham B.L., Gerry A.C. (2012). Northern fowl mite (*Ornithonyssus sylviarum*) control evaluations using liquid formulations of diatomaceous earth, kaolin, sulfur, azadirachtin, and *Beauveria bassiana* on caged laying hens. J. Appl. Poult. Res..

[126] Martin C.D., Mullens B.A. (2012). Housing and dustbathing effects on northern fowl mites (*Ornithonyssus sylviarum*) and chicken body lice (*Menacanthus stramineus*) on hens. Med. Vet. Entomol..

[127] Murillo A.C., Mullens B.A. (2016). Timing diatomaceous earth-filled dustbox use for management of northern fowl mites (Acari: Macronyssidae) in cage-free poultry systems. J. Econ. Entomol..

[128] Showler T.A., Flores N., Caesar R.M., Mitchel R.D., Perez de Leon A.A.P. (2020). Lethal Effects of a Commercial Diatomaceous Earth Dust Product on *Amblyomma americanum* (Ixodida: Ixodidae) Larvae and Nymphs. J. Med. Entomol..

[129] Dawson R.D. (2004). Efficacy of diatomaceous earth at reducing populations of nest-dwelling ectoparasites in tree swallows. J. Field Ornithol..

[130] Kilpinen O., Steenberg T. (2009). Inert dusts and their effects on the poultry red mite (*Dermanyssus gallinae*). Exp. Appl. Acarol..

[131] Brinkman M.A., Gardner W.A. (2001). Use of diatomaceous earth and entomopathogen combinations against the red imported fire ant (Hymenoptera: Formicidae). Florida Entomol..

[132] Constanski K.C., Zorzetti J., Santoro P.H., Hoshino A.T., Oliveira Janiero Neves P.M. (2016). Inert powders alone or in combination with neem oil for controlling *Spodoptera eridania* and *Spodoptera frugiperda* (Lepidoptera: Noctuidae) larvae. Semin. Agrar..

[133] Singh B., Singh V. (2016). Laboratory and field studies demonstrating the insecticidal potential of diatomaceous earth against wheat aphids in rice-wheat cropping system of Punjab (India). Cereal Res. Commun..

[134] Aniwanou C.T.S., Sinzogan A.A.C., Deguenon J.M., Sikirou R., Stewart D.A., Ahanchede A. (2021). Bio-efficacy of diatomaceous earth, household soaps, and neem oil against *Spodoptera frugiperda* (Lepidoptera: Noctuidae) larvae in Benin. Insects.

[135] Constantinescu-Aruxandei D., Lupu C., Oancea F. (2020). Siliceous Natural Nanomaterials as Biorationals—Plant Protectants and Plant Health Strengtheners. Agronomy.

[136] Khaled W., Ben Fekih I., Harbaoui I., Boukhris-Bouhachem S. Insecticidal activity assessment of Thymus capitatus essential oils in combination with natural abrasives against Myzus persicae. Proceedings of the Second Africa-International Allelopathy Congress.

[137] Lozano-Contreras M.G., Maldonado-Blanco M.G., Elías-Santos M., González-Hernández A., Nava-Camberos U. (2013). Application of *Isaria fumosorosea* blastospores produced in liquid culture for control of *Bemisia argentifolii* on cotton plants. Southwest Entomol..

[138] Ulrichs C.H., Mewis I., Schnitzler W.H. (2001). Efficacy of neem and diatomaceous earth against cowpea aphids and their deleterious effect on predating Coccinelidae. J. Appl. Entomol..

[139] El-Wakeil N.E., Saleh S.A. (2009). Effects of neem and diatomaceous earth against *Myzus persicae* and associated predators in addition to indirect effects on artichoke growth and yield parameters. Arch. Phytopathol. Plant Prot..

[140] Shah R., Appleby M. (2019). Testing the contact and residual toxicity of selected low-risk pesticides to *Tetranychus Urticae* Koch and its Predators. Sarhad J. Agric..

[141] Adams C.T., Banks W.A., Lofgren C.S. (1988). Red Imported Fire Ant (Hymenoptera: Formicidae): Correlation of Ant Density with Damage to Two Cultivars of Potatoes (*Solanum tuberosum* L.). J. Econ. Entomol..

[142] Hagstrum D.W., Phillips T.W. (2017). Evolution of stored-product entomology: Protecting the world food supply. Annu. Rev. Entomol..

[143] Parkin E.A. (1944). Control of the granary weevil with finely ground mineral dusts. Ann. Appl. Biol..

[144] Korunić Z., Cenkowski S., Fields P.G. (1998). Grain bulk density as affected by diatomaceous earths and application method. Postharvest Biol. Technol..

[145] Jackson K., Webley D., Highley E., Wright E.J., Banks H.J., Champ B.R. (1994). Effects of Dryacide on the Physical Properties of Grains, Pulses and Oilseeds. Stored Product Protection, Proceedings of the Sixth International Conference on Stored Product Protection, Canberra, Australia, 1994.

[146] Gesraha M.A., Ebeid A.R., Salem N.Y.M., Abdou W.L. (2017). Comparative Study on Some Biological Indices of *Agrotis ipsilon* (Lepidoptera: Noctuidae) Larvae Treated with Three Control Agents under Laboratory Conditions. Annu. Res. Rev. Biol..

[147] Ferreira-Filho P.J., Wilcken C.F., Neves D.A., Pogotto M.H.F.A.D., Carmo J.B., Guerreiro J.C., Serrao J.E., Zanuncio J.C. (2015). Does Diatomaceous Earth Control Leaf-Cutter Ants (Hymenoptera: Formicidae) in the *Eucalyptus* Plantations?. J. Ec. Entomol..

[148] Mucha-Pelzer T., Debnath N., Goswami A., Mewis I., Ulrichs C. (2008). Bekämpfung von *Epilachna vigintioctopunctata* (F.) und Spodoptera litura (F.) mit Silikaten. Gesunde Pflanzen.

[149] Mitchell R.D., Mott D.W., Dhammi A., Reisig D.D., Roe R.M. Field Evaluation of a New Thrips Control Agent for Cotton: A Mechanical Insecticide. Proceedings of the Beltwide Cotton Conferences.

[150] Ebadollahi A., Sadeghi R. (2018). Diatomaceous Earth and Kaolin as Promising Alternatives to the Detrimental Chemicals in the Management of *Spodoptera exigua*. J. Entomol..

[151] Desmarchelier J.M., Dines J.C. (1987). Dryacide treatment of stored wheat: Its efficacy against insects, and after processing. Aust. J. Exp. Agric..

[152] Aldryhim Y.N. (1990). Efficacy of the amorphous silica dust, Dryacide, against *Tribolium confusum* Duv. and *Sitophilus granarius* (L.) (Coloptera: Tenebrionidae and Curculionidae). J. Stored Prod. Res..

[153] McLaughlin A., Highley E., Wright E.J., Banks H.J., Champ B.R. (1994). Laboratory Trials on Desiccant Dust Insecticides. Stored Product Protection, Proceedings of the Sixth International Conference on Stored Product Protection.

[154] Subramanyam B.H., Hagstrum D.W., Subramanyam B.H., Hagstrum D.W. (1995). Resistance Measurement and Management. Integrated Management of Insects in Stored Products.

[155] Musser F.R., Shelton A.M. (2005). The influence of post-exposure temperature on the toxicity of insecticides to *Ostrinia nubilalis* (Lepidoptera: Crambidae). Pest Manag. Sci..

[156] Khan H.A.A., Akram W. (2014). The effect of temperature on the toxicity of insecticides against *Musca domestica* L.: Implications for the effective management of diarrhea. PLoS ONE.

[157] Fields P., Dowdy A., Marcotte M. Structural Pest Control: The Use of an Enhanced Diatomaceous Earth Product Combined with Heat Treatment for the Control of Insect Pests in Food Processing Facilities. Canada–United States Working Group on Methyl Bromide Alternatives. 1997. http://res.agr.ca/winn/Heat-DE.htm.

[158] Dowdy A.K. (1999). Mortality of red four beetle, *Tribolium castaneum* (Coleoptera: Tenebrionidae) exposed to high temperature and diatomaceous earth combinations. J. Stored Prod. Res..

[159] Dowdy A.K., Fields P.G. (2002). Heat combined with diatomaceous earth to control the confused flour beetle (Coleoptera: Tenebrionidae) in a flour mill. J. Stored Prod. Res..

[160] Cook D.A., Collins D.A., Collins L.E. (2004). Efficacy of diatomaceous earths, applied as structural treatments, against stored product insects and mites. HGCA Proj. Re..

[161] Collins D.A., Cook D.A. (2006). Laboratory evaluation of diatomaceous earths, when applied as dry dust and slurries to wooden surfaces, against stored-product insect and mite pests. J. Stored Prod. Res..

[162] Collins D.A., Cook D.A. (2006). Laboratory studies evaluating the efficacy of diatomaceous earths, on treated surfaces, against stored-product insect and mite pests. J. Stored Prod. Res..

[163] Schöller M., Reichmuth C. (2010). Field trials with the diatomaceous earth SilicoSec^®^ for treatment of empty rooms and bulk grain. Julius-Kühn-Archiv.

[164] Ertürk S., Atay T., Toprak U., Alkan M. (2020). The efficacy of different surface applications of wettable powder formulation of Detech® diatomaceous earth against the rice weevil, *Sitophilus oryzae* (L.) (Coleoptera: Curculionidae). J. Stored Prod. Res..

[165] Gowers S.L., Le Patourel G.N.J. (1984). Toxicity of deposits of an amorphous silica dust on different surfaces and their pick-up by *Sitophilus granarius* (Coleoptera: Curculionidae). J. Stored Prod. Res..

[166] Cook D.A. (2003). The Efficacy of High Temperature and Diatomaceous Earth Combinations Against Adults of the Red Flour Beetle Tribolium Castaneum (Coleoptera: Tenebrionidae) and the Grain Weevil Sitophilus Granarius (Coleoptera: Curculionidae). Proceedings of the BCPC International Congress—Crop Science and Technology.

[167] Cox P.D., Parish W.E. (1991). Effects of refuge content and food availability on refuge-seeking behavior in *Cryptolestes ferrugineus* (Stephens) (Coleoptera: Cucujdiae). J. Stored Prod. Res..

[168] Vrba C.H., Arai H.P., Nosal M. (1983). The effect of silica aerogel on the mortality of *Tribolium confusum* (Duval) as a function of exposure time and food deprivation. Can. J. Zool..

[169] Loschiavo S.R. (1988). Availability of food as a factor in the effectiveness of a silica aerogel against the merchant grain beetle (Coleoptera: Cucujidae). J. Econ. Entom..

[170] White N.D.G., Loschiavo S.R. (1989). Factors affecting survival of the merchant grain beetle (Coleoptera: Cucujidae) and the confused flour beetle (Coleoptera: Tenebrionidae) exposed to silica aerogel. J. Econ. Entom..

[171] Bridgeman B., Zuxun J., Quan L., Yongsheng L., Xianchang T., Lianghua G. Application technology and usage patterns of diatomaceous earth in stored product protection. Stored-product Protection. Proceedings of the Seventh International Working Conference on Stored-product Protection.

[172] Banks J.H., Fields P.G., Jayas D.S., White N.D.G., Muir W.E. (1995). Physical Methods for Insect Control in Stored-Grain Ecosystems. Stored-Grain Ecosystems.

[173] Golob P. (1997). Current status and future perspective for inert dusts for control of stored product insects. J. Stored Prod. Res..

[174] Kljajić P., Andrić G., Adamović M., Bodroža-Solarov M., Marković M., Perić I. (2010). Laboratory assessment of insecticidal effectiveness of natural zeolite and diatomaceous earth formulations against three stored-product beetle pests. J. Stored Prod. Res..

[175] Kljajić P., Andrić G., Milan A., Golić M.P. (2011). Possibilities of application of natural zeolites in stored wheat grain protection against pest insects. J. Process. Energy Agric..

[176] Bodroža-Solarov M., Kljajić P., Andrić G., Filipčev B., Šimurina O., Golić P.M., Adamovic M. (2011). Application of principal component analysis in assessment of relation between the parameters of technological quality of wheat grains treated with inert dusts against rice weevil (*Sitophilus oryzae* L.). Pestic. Fitomed..

[177] Rumbos C.I., Sakka M., Berillis P., Athanassiou C.G. (2016). Insecticidal potential of zeolite formulations against three stored grain insects, particle size effect, adherence to kernels and influence on test weight of grains. J. Stored Prod. Res..

[178] Floros G., Kokkari A., Kouloussis N., Kantiranis N., Damos P., Filippidis A., Koveos D. (2017). Evaluation of the natural zeolite lethal effects on adults of the bean weevil under different temperatures and relative humidity regimes *J*. Econ. Entom..

[179] Westwood G.S., Huang S.W., Keyhani N.O. (2006). Molecular and immunological characterization of allergens from the entomopathogenic fungus *Beauveria bassiana*. Clin. Mol. Allergy..

[180] Chen C.Y., Chen M.F. (2013). Susceptibility of field populations of the lesser grain borer, *Rhyzopertha dominica* (F.), to deltamethrin and spinosad on paddy rice in Taiwan. J. Stored Prod Res..

[181] Daglish G.J., Nayak M.K. (2018). Prevalence of resistance to deltamethrin in *Rhyzopertha dominica* (F.) in eastern Australia. J. Stored Prod Res..

[182] Nayak M.K., Daglish G.J., Phillips T.W., Ebert P.R. (2020). Resistance to the fumigant phosphine and its management in insect pests of stored products: A global perspective. Ann. Rev. Entomol..

